# Effects of Varying Protein Amounts and Types on Diet-Induced Thermogenesis: A Systematic Review and Meta-Analysis

**DOI:** 10.1016/j.advnut.2024.100332

**Published:** 2024-10-31

**Authors:** Liana L Guarneiri, Caryn G Adams, Bibiana Garcia-Jackson, Katie Koecher, Meredith L Wilcox, Kevin C Maki

**Affiliations:** 1 Midwest Biomedical Research, Addison, IL, United States; 2 Bell Institute of Health and Nutrition, General Mills Inc, Minneapolis, MN, United States; 3 Department of Applied Health Science, Indiana University School of Public Health-Bloomington, Bloomington, IN, United States

**Keywords:** metabolism, protein, diet-induced thermogenesis, thermic effect of food, energy expenditure, substrate utilization, fat oxidation

## Abstract

Protein is the most thermogenic macronutrient, but it is unclear how different amounts and types of protein impact diet-induced thermogenesis (DIT). The purpose of this meta-analysis was to compare the impact of isocaloric meals/diets containing different amounts or types of protein on energy metabolism. Databases were searched in June 2024 for studies that compare DIT or total daily energy expenditure (TDEE) in response to isocaloric acute meals or longer-term diets containing different amounts or types of protein. After identifying 3894 records, 52 studies were included. Standardized mean difference (SMD) estimates and 95% confidence intervals (CIs) were calculated for each outcome. In acute studies, intake of higher compared with lower-protein meals resulted in greater DIT (SMD: 0.45; 95% CI: 0.26, 0.65; *P* < 0.001) and TDEE (SMD: 0.52; 95% CI: 0.30, 0.73; *P* < 0.001). Notably, the subgroup analysis indicated that this effect on DIT was statistically significant for studies involving participants with normal weight but not overweight/obesity, although it is not clear if this finding was a true effect or because of study design characteristics. In chronic studies (ranging from 4 d to 1 y), intake of higher compared with lower-protein diets resulted in greater TDEE (SMD: 0.29; 95% CI: 0.10, 0.48; *P =* 0.003) and resting energy expenditure (SMD: 0.18; 95% CI: 0.01, 0.35; *P =* 0.039), but no differences in DIT (SMD: 0.10; 95% CI: –0.08, 0.28; *P =* 0.27). There was no evidence that different types of protein impacted energy metabolism. Higher protein meals/diets increase components of energy expenditure.

This trial was registered at the International Prospective Register of Systematic Reviews (https://www.crd.york.ac.uk/prospero; PROSPERO 2023) as CRD42023389642.

**Statement of significance**

This was the first meta-analysis to assess the effect of acute and longer-term intakes of protein on diet-induced thermogenesis.

## Introduction

More than 40% of adults in the United States have obesity [1], which increases risk for chronic diseases such as cardiovascular disease, diabetes, kidney disease, osteoarthritis, and cancer [2]. For many individuals, obesity develops slowly over decades. Researchers estimate that an excess intake of 10–20 kcal/d contributes to the average yearly weight gain of 0.5–1 kg among United States adults, and this persistent annual weight gain contributes to obesity over time [[3], [4], [5], [6]]. Therefore, it is feasible that small adjustments in energy intake or energy expenditure may prevent the energy imbalances that are fueling annual weight gain and subsequent obesity.

Diet-induced thermogenesis (DIT) is the energy expended during the digestion, absorption, and storage of food, and it contributes to ∼10% of total daily energy expenditure (TDEE) [7,8]. Suppression of energy metabolism, including DIT, may be associated with obesity [9,10]. It is generally accepted that protein is the most thermogenic macronutrient [11,12]. Two systematic reviews have investigated the effects of higher protein compared with lower-protein acute meals on DIT, and the authors of both reviews concluded that higher protein intake elicits a higher thermogenic response [13,14]. Regarding longer-term trials, Wycherly et al. conducted a systematic review and meta-analysis of 4 randomized, controlled trials that compared higher-protein with lower-protein weight loss diets and concluded that higher-protein diets mitigate reductions in resting energy expenditure (REE) during weight loss [142.3 kcal/d; 95% confidence interval (CI): 67.0, 1124.1], but DIT was not evaluated [15]. Consuming protein-rich meals and diets may partially or fully offset daily energy imbalances and thus influence changes in body weight and composition, but no meta-analysis has investigated the effect of acute and longer-term intakes of protein on DIT. Therefore, the purpose of this systematic review and meta-analysis was to examine the impact of different amounts and types of protein intake on DIT.

## Methods

The systematic review and meta-analysis followed the PRISMA guidelines and was prospectively registered in the PROSPERO database (CRD42023389642).

### Search strategy

PubMed and Web of Science were searched for studies from inception to June 2024. Food-related search words included macronutrient, diet composition, isoenergetic meal, macronutrient composition, carbohydrate, fat, dietary fat, high-protein, low-protein, high-carbohydrate, low-carbohydrate, high-fat, low-fat, isocaloric meal, protein, dietary protein, pea, whey, casein, soy, soybean, tofu, chicken, beef, pork, meat, fish, seafood, dairy, milk, cheese, egg, walnut, peanut, hazelnut, almond, pistachio, cashew, macadamia, pecan, pine nut, Brazil nut, mixed nut, texturized vegetable protein, tempeh, miso, protein supplement, and legume. Study design search words included controlled clinical trial, clinical trial, clinical research study, open label, open-label, non-randomized, nonrandomized, quasi-experimental, and quasiexperimental. Energy metabolism search words included energy expenditure, total energy expenditure, diet induced thermogenesis, diet-induced thermogenesis, dietary thermogenesis, thermic effect of feeding, meal-induced thermogenesis, specific dynamic action, thermic effect of food, thermogenic effect of food, meal-induced thermogenesis, postprandial metabolism, postprandial metabolic rate, postprandial thermogenesis, respiratory quotient (RQ), fat oxidation, protein oxidation, carbohydrate oxidation, DIT, TEF, TDEE, and RQ. Limits were applied to include only studies in English. The reference lists from included articles were also reviewed to identify additional relevant studies to include.

### Inclusion and exclusion criteria

To be included, the studies had to be peer-reviewed, published in English, and a prospective randomized or nonrandomized controlled trial (parallel or crossover). In addition, the studies needed to compare DIT or TDEE in response to isocaloric acute meals or longer-term diets containing different amounts or types of protein. For studies comparing the amount of protein, the percent energy from protein had to be ≥5% different between the higher- and lower-protein meals. For acute meal studies, only mixed meals were included; thus, meals had to contain ≥5% of energy each from protein, carbohydrate, and fat. Meals that contained alcohol were not included. For studies that involved 3 or more meals/diets, if 1 or more meals/diets did not meet inclusion criteria, then data from the other meals/diets were extracted (as long as both an active and control/referent meal/diet met inclusion criteria). We included metabolism measurements from doubly labeled water, indirect calorimetry systems, and metabolic chambers. If an article reported metabolism results separately for multiple meals in 1 d, data from the first meal of the day were extracted. Participants in included studies were generally healthy adults or children with or without stable metabolic conditions such as overweight/obesity, prediabetes, type 2 diabetes, metabolic syndrome, and hypercholesterolemia. The minimum length of postprandial metabolism measurements was ≥2 h.

**FIGURE 1:**
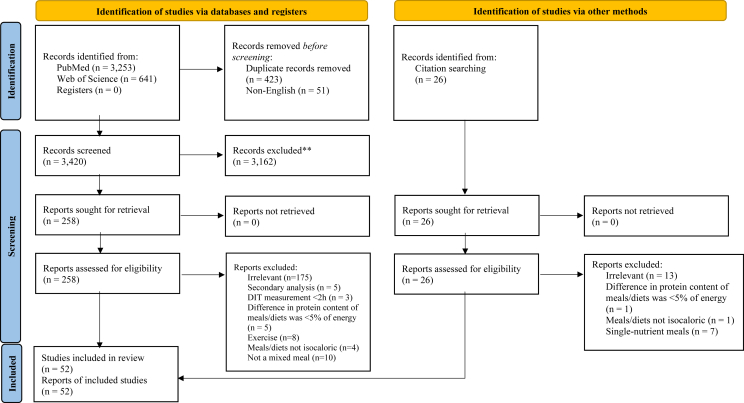
PRISMA flow diagram. The authors excluded 847 records, and Abstrackr (a semiautomated tool for predictive title and abstract screening) excluded 2040 records.

Studies involving the following populations were excluded: pregnant/lactating women, infants, bariatric, rare conditions, genetic disorders, and severe chronic disease (chronic kidney disease, hepatic disease, cancer, neurodegenerative disease, etc.). For studies that reported energy metabolism results for both included and excluded populations in separate groups (for example, pregnant compared with nonpregnant women), only the data from the group that met inclusion criteria were extracted. Exclusion criteria also included the following: *1*) reviews, editorials, and expert opinions; *2*) studies using observational, retrospective, case-control, and single-arm study designs; *3*) studies using animal models; *4*) studies in which exercise occurred during the metabolism measurement; and *5*) studies in which meals were administered as enteral, intranasal, or intragastric infusion instead of orally. For studies that reported energy metabolism results for both resting and exercise conditions, only the data for the resting conditions were extracted. In cases when multiple secondary publications of the same data set were identified, the publication with the most complete data for energy metabolism was used. The study selection process is illustrated in Figure 1.

### Study selection

After the systematic search of PubMed and Web of Science, individual studies were screened based on the title and abstracts for the inclusion and exclusion criteria using Abstrackr, a semiautomated tool for predictive title and abstract screening. Rathbone et al. [16] compared Abstrackr with manual screening of citations in 4 systematic reviews and reported that the percentage of citations missed by Abstrackr that were included in the published reviews ranged from 0% to 0.21%, indicating almost complete reliability. Next, the full texts of studies were reviewed. Studies that met the inclusion criteria during the full-text review were included in the systematic review and meta-analysis.

### Data extraction

The following study details were recorded: first author, year of publication, title, journal, location, study design, participant characteristics, metabolism measurement method and duration, duration of chronic diets, acute meal/chronic diet nutrient compositions, and sample size. Additionally, the mean, SD, SE, and/or 95% CIs were recorded for DIT, resting metabolic rate (RMR), TDEE, and postprandial substrate utilization [carbohydrate oxidation, fat oxidation, and respiratory exchange ratio (RER)]. RQ was considered interchangeable with RER, and REE was considered interchangeable with RMR. Physical activity data generated from questionnaires were not extracted. A risk of bias analysis was conducted using the Cochrane risk of bias tool for randomized studies [17] and the Risk of Bias In Non-randomized Studies of Interventions (ROBINS-I) tool for the nonrandomized study [18]. The quality of evidence was assessed using the Grading or Recommendations Assessment, Development and Evaluation (GRADE) method [19]. Data were extracted and assessed for bias by 1 author (LLG) and verified for accuracy by a second author (CGA). When studies reported findings from multiple models, results from the model that adjusted for the greatest number of covariates were extracted. When a longer-term study assessed the outcomes at multiple follow-up periods, data were extracted for the longest follow-up period. When studies tested acute meals and longer-term diets, data were extracted for both. When studies tested multiple active or referent/control conditions (for example, the same condition at various dose levels), data for all conditions were extracted. When studies reported metabolism results with all participants together and by weight status, data for all participants together were extracted.

### Statistical methods

Standardized mean difference (SMD) estimates and 95% CIs were calculated for each outcome. Statistical significance for individual study and pooled estimates were declared when the 95% CI did not include the null value of 0 (that is, *P* < 0.05). The prespecified primary outcome was the SMD between the active and control/referent conditions for the DIT after acute meals and/or longer-term diets containing varying types or amounts of protein. The secondary outcomes are the SMD between the active and control/referent conditions for TDEE, sleeping metabolic rate, activity energy expenditure, and fasting/postprandial substrate utilization. Based on the characteristics of the dataset, an a priori decision was made on which studies would be pooled together for the analysis looking at the effect of different types of protein. The following analyses were included: *1*) diets containing animal and vegetable proteins compared with diets containing vegetable protein only, *2*) acute meals containing whey compared with no whey, and *3*) acute meals containing fish compared with red meat.

Analyses were conducted using R Statistical Software (v4.3.0) [20]. Meta-analyses were performed using the meta R package (v6.5-0) [21]. SMDs were computed as the mean difference in the change from baseline to post-condition divided by the pooled SD. Mean changes were computed, when necessary, from means for baseline, end-of-condition, or percent change. When mean changes could not be computed, means for percent change were used followed by means for end-of-condition. Das et al. [22] reported medians and interquartile range (IQR) limits for the percent changes in TDEE, REE, and RER. Means and SDs were approximated using the medians and IQR/1.35, respectively [23]. If a study reported means or medians but no measure of variability, missing SDs were extrapolated separately for each condition using the maximum coefficient of variation from similar (active or chronic) studies. Paired analyses were approximated for crossover studies using the methods described in section 23.2.7 of the Cochrane Handbook and assuming a correlation coefficient of 0.59 [[23], [24]]. Multiarm studies with 2 or more correlated comparisons (that is, when participants contribute data to >1 relevant comparison) were included in the analysis by first computing an average of the effects of the correlated comparisons and a variance for the study assuming a between-comparison correlation of 0.59. The average SMD and study-wide variance were then used as a single effect for the study in the pooled analysis [25]. Analytical sample sizes reported hereinafter refer to the number of comparisons included in the analysis after pooling effect sizes for correlated, within-study comparisons.

Additionally, an a priori decision was made to use a random-effects model as the primary approach based on the characteristics of the dataset. Individual studies were weighted based on the inverse of its variance and using the DerSimonian–Laird method for estimation of between-study heterogeneity. Heterogeneity was assessed using Cochran’s *Q* and the *I*^2^ statistic (low heterogeneity: 0%–40%; moderate/higher heterogeneity: >40%) [23]. A metaregression was performed to evaluate the dose–response relationship of different amounts of protein on DIT. The presence of small study effects was assessed visually by examining funnel plots, as well as statistically by using Egger’s regression method [23]. Potential publication bias was assessed by examining contour-enhanced funnel plots for missing studies in areas that correspond to statistically nonsignificant or unfavorable results.

Subgroup analyses were performed on all energy metabolism outcomes for protein difference, sex, BMI, weight status, age, study design, risk of bias, and blinding. The BMI subgroup categorized each study based on whether the mean BMI for the given study was greater than or equal to the median BMI for all studies or below the median BMI for all studies. The weight status subgroup categorized studies based on whether the authors reported if they were recruiting participants with underweight, normal weight, overweight, obesity, or a combination of these categories. Additionally, for substrate utilization outcomes, subgroup analyses were performed for carbohydrate difference. The protein difference represents the difference in the percent of energy from protein in the higher-protein group minus the lower-protein group. The carbohydrate difference represents the difference in percent of energy from carbohydrate in the lower-protein group from the higher-protein group. For energy outcomes in chronic studies, subgroup analyses investigating the effect of energy balance (hypocaloric, eucaloric compared with hypercaloric diets) and intervention lengths were also completed. For DIT, an additional subgroup analysis evaluated the effect of consuming >1 meal during the metabolism measurement. Based on the results of the BMI subgroup analysis evaluating DIT in acute studies, the same subgroup analysis was conducted in crossover studies only, and sensitivity analyses were conducted in groups of studies with similar study design characteristics. No subgroup analyses were conducted for studies that investigated the effect of different types/sources of protein on energy metabolism because of the limited number of studies. Correlated, within-study comparisons that fell in the same subgroup were pooled before running each subgroup analysis. Subgroup analyses were not performed for subgroups with fewer than 3 comparisons after addressing multiple within-study comparisons.

## Results

The database searches resulted in 3894 results, and 423 duplicates and 51 non-English articles were removed. After reviewing titles and abstracts, 3162 studies were excluded, and 210 were excluded after a full-text review. Forty-eight studies were included from the database searches, and an additional 4 studies were identified when searching reference lists. Therefore, 52 studies (51 randomized and 1 nonrandomized) were included in the final meta-analysis, which represented 1232 participants (Table 1) [[26], [27], [28], [29], [30], [31], [32], [33], [34], [35], [36], [37], [38], [39], [40], [41], [42], [43], [44], [45], [46], [47], [48], [49], [50], [51], [52], [53], [54], [55], [56], [57], [58], [59], [60], [61], [62], [63], [64], [65], [66], [67], [68], [69], [70], [71], [72], [73], [74], [75], [76], [77]]. No studies were excluded from the analysis because of incomplete reporting.

**TABLE 1:** Characteristics of included studies.

First author, year	Study design	Population	Total no. of subjects in analysis (*n*)	Duration of metabolism measurement (h)	Chronic intervention duration (d)	Intervention details	Control/referent details	% Difference in energy from protein between intervention and control/referent
Acheson et al. (2011)^1^ [31]	Randomized, crossover	Healthy adults with normal weights	23	5.5	N/A	Whey meal: subjects consumed an acute meal containing BiPro whey protein isolate.		N/A
						Casein meal: subjects consumed an acute meal containing MPI 85 MC micellar casein.		
						Soy meal: subjects consumed an acute meal containing Supro soy protein isolate.		
Barnard et al. (2005) [54]	Randomized, parallel	Healthy adults with BMIs of 26–44 kg/m^2^	59	3	98	Low-fat vegan diet: subjects consumed a diet containing vegetables, fruits, grains, and legumes. A liquid meal (Boost Plus) was consumed at baseline and 14 weeks for the measurement of DIT.		N/A
						NCEP Step II diet: subjects followed former NCEP Step II guidelines (including ≤7% energy from saturated fat and <200 mg/d cholesterol). A liquid meal (Boost Plus) was consumed at baseline and 14 weeks for the measurement of DIT.		
Batterham et al. (2008)^1^ [72]	Randomized, crossover	Healthy adults	18	8	N/A	High-protein (low GI) meals: subjects consumed meals containing a cheese and tomato omelet, bacon, open sandwich with beef, coffee, bread, margarine, chutney, salad, and low-fat yogurt.	Lower-protein meals: subjects consumed meals containing a cheese sandwich, rice pudding, cornflakes, sugar, milk, coffee, bread, margarine, jam, and pesto.	21
Bellissimo et al. (2020) [55]	Randomized, crossover	Healthy adolescents (9–14 y) that habitually consume breakfast	9	5	N/A	Protein meal: subjects consumed an acute meal containing egg, butter, cheese, home fries, and ketchup.	Carbohydrate meal: subjects consumed an acute meal containing white bread, butter, and jam.	34
Bendtsen et al. (2014) [42]	Randomized, crossover	Healthy adults (22–40 y) with BMIs of 27–35 kg/m^2^	24	24	N/A	Hydrolyzed casein meals: subjects consumed protein shakes containing hydrolyzed casein inside a respiration chamber.		
						Intact casein meals: subjects consumed protein shakes containing intact casein with all meals inside respiration chamber.		
						Intact whey meals: subjects consumed protein shakes containing intact whey inside a respiration chamber.		
Bottin et al. (2016) [68]	Randomized, crossover	Healthy adults (18–65 y) with BMIs of 25–32 kg/m^2^	14	3	N/A	Mycoprotein meal: subjects consumed a meal containing mushroom risotto, vegetables, rice, cheese, and pesto.		N/A
						Chicken meal: subjects consumed a meal containing chicken risotto, vegetables, rice, cheese, and pesto.		
Bray et al. (2012) [41]	Randomized, parallel	Healthy adults (20–70 y) with BMIs of 25–40 kg/m^2^	89	N/A^2^	182	Higher-protein diet: participants received dietary counseling to consume 25% of total energy from protein and to achieve a 750 kcal/d deficit. DLW measurements were completed at baseline and 6 mo.	Lower-protein diet: participants received dietary counseling to consume 15% of total energy from protein and to achieve a 750 kcal/d deficit. DLW measurements were completed at baseline and 6 mo.	10
Bray et al. (2015) [63]	Randomized, parallel	Healthy adults (18–35 y) with BMIs 19.7–29.6 kg/m^2^	25	24	56	Higher-protein diet: subjects consumed a higher-protein hypercaloric diet. TDEE was measured in a respiration chamber on days 1, 14, and 56 of overfeeding.	Lower-protein diet: subjects consumed a low-protein hypercaloric diet. TDEE was measured in a respiration chamber on days 1, 14, and 56 of overfeeding.	20
						Normal-protein diet: subjects consumed a normal-protein hypercaloric diet. TDEE was measured in a respiration chamber on days 1, 14, and 56 of overfeeding.		10
Brehm et al. (2005) [62]	Randomized, crossover	Healthy women (≥18 y) with BMIs of 30–35 kg/m^2^	8	5	N/A	Higher-protein meal: subjects consumed an acute meal containing pork sausage, liquid eggs, margarine, cheddar cheese, and nonfat milk.	Lower-protein meal: subjects consumed an acute meal containing pancakes, maple syrup, banana, margarine, and nonfat milk.	15
Bronstein et al. (1995)^1^ [69]	Randomized, crossover	Healthy adults with normal weights or overweight	17	4	N/A	Higher-protein meal: subjects consumed scrambled eggs, toast, orange juice, butter, and jam.	Lower-protein meal: subjects consumed polycose (glucose polymers), orange juice, toast, butter, and jam.	10
Crovetti et al. (1998) [29]	Randomized, crossover	Healthy women with normal weights	10	7	N/A	Protein meal: subjects consumed an acute meal comprising bresaola and crackers.	Carbohydrate meal: subjects consumed an acute meal comprising pasta, tomato sauce, and olive oil.	58
							Fat meal: subjects consumed an acute meal comprising mascarpone and crackers.	60
Das et al. (2008) [75]	Randomized, parallel	Healthy adults (24–42 y)	29	N/A^2^	365	Higher-protein (low GI) diet: subjects consumed a higher-protein hypocaloric diet. DLW measurements were completed at baseline, 3, 6, and 12 mo.	Lower-protein (high GI) diet: subjects consumed a lower-protein hypocaloric diet. DLW measurements were completed at baseline, 3, 6, and 12 mo.	10
Gentile et al. (2015) [39]	Randomized, crossover	Healthy women	16	3	N/A	Protein meal: subjects consumed pancakes prepared with whey protein and waxy maize starch or resistant starch.	Carbohydrate meal: subjects consumed pancakes prepared with waxy maize starch or resistant starch.	12
Hochstenbach-Waelen et al. (2009) [73]	Randomized, crossover	Healthy adults with BMIs of 20–33 kg/m^2^	23	35	5	Higher-protein diet: subjects consumed a higher-protein diet comprising normal food products and various protein sources for 3 d. Next, subjects consumed a higher-protein diet comprising mostly of custard containing casein or gelatin during a 36-h stay in a respiration chamber.	Lower-protein diet: subjects consumed a lower-protein diet comprising normal food products and various protein sources for 3 d. Next, subjects consumed a lower-protein diet comprising mostly of custard containing casein or gelatin during a 36-h stay in a respiration chamber.	15
Hursel et al. (2010) [53]	Randomized, crossover	Healthy adults	35	4	N/A	Whey meal: subjects consumed a higher-protein yogurt drink with added total whey protein.	Whole milk meal: subjects consumed a normal-protein yogurt drink that contained whole milk.	26
						α-lactalbumin meal: subjects consumed a higher-protein yogurt that contained caseinomacropeptide-depleted α-lactalbumin-enriched whey protein.		26
Jacobsen et al. (2005)^1^ [67]	Randomized, crossover	Healthy adults (18–50 y) that were moderately overweight	10	24	7	Higher-protein (higher-calcium) diet: subjects consumed a higher-protein, higher-calcium diet for 7 d. Meals were consumed inside a respiration chamber on days 1 and 7 of the diet.	Normal-protein (higher-calcium) diet: subjects consumed a normal-protein, higher-calcium diet for 7 d. Meals were consumed inside a respiration chamber on days 1 and 7 of the diet.	8
Johnston et al. (2002) [27]	Randomized, crossover	Healthy young adults (19–22 y) with normal weights	10	2.5	N/A	Protein meal: subjects consumed an acute meal containing egg white, cottage cheese, turkey, and tuna.	Carbohydrate meal: subjects consumed an acute meal in which grains were substituted for protein foods.	13
Kassis et al. (2019)^1^ [38]	Randomized, crossover	Healthy adults (20–45 y) with BMIs of 25–32 kg/m^2^ and %BF in or above the overweight range	17	5.5	N/A	Higher-protein whey meal: subjects consumed a commercial protein drink containing 50 g whey.	Lower-protein whey meal: subjects consumed a commercial protein drink containing 30 g whey.	27
						Higher-protein casein meal: subjects consumed a commercial protein drink containing 50 g casein.		26
Labayen et al. (2004) [71]	Randomized, parallel	Healthy women (20–50 y) with BMIs <25 or >30 kg/m^2^	26	6	N/A	Higher-protein meal: subjects consumed a higher-protein liquid meal replacement (Modifast).	Lower-protein meal: subjects consumed a lower-protein liquid meal replacement (Meritene).	15
Leidy et al. (2007)^3^ [70]	Randomized, crossover	Healthy women (≥21 y) with BMIs of 26–37 kg/m^2^	38	4	84	Higher-protein diet: subjects consumed a higher-protein hypocaloric diet for 12 weeks. During week 9, subjects consumed a higher-protein and a normal-protein meal on 2 separate days for the measurement of DIT.	Lower-protein diet: subjects consumed a lower-protein hypocaloric diet for 12 weeks. During week 9, subjects consumed a higher-protein and a normal-protein meal on 2 separate days for the measurement of DIT.	11
Lejeune et al. (2006) [56]	Randomized, crossover	Healthy women (18–40 y) with BMIs of 20–25 kg/m^2^	12	36	N/A	Higher-protein meals: subjects consumed meals containing chicken, soy milk, tuna, ham, yogurt, milk, merengue, feta cheese, rice, bread, butter, vegetables, soup, and muesli.	Lower-protein meals: subjects consumed meals containing chicken, soy dessert, tuna, cottage cheese, bread, orange juice, fruits, vegetables, butter, soup, and muesli.	20
Li et al. (2016) [51]	Randomized Parallel study design for the comparison of the omnivorous with lacto-ovo vegetarian diets Crossover study design for the comparison of different protein quantities within each diet	Healthy adults (≥21 y) with BMIs of 27–36.9 kg/m^2^	34	4	28	Omnivorous meal/diet: subjects consumed a hypocaloric omnivorous diet for 3 consecutive 4-week periods varying in protein quantity (10%, 20%, or 30% of total energy). Acute meals reflected the chronic diet and were consumed on day 28 of each diet for the measurement of DIT.		10–20
						Lacto-ovo vegetarian meal/diet: subjects consumed a hypocaloric lacto-ovo vegetarian diet for 3 consecutive 4-week periods varying in protein quantity (10%, 20%, or 30% of total energy). Acute meals reflected the chronic diet and were consumed on day 28 of each diet for the measurement of DIT.		10–20
Lorenzen et al. (2012) [36]	Randomized, crossover	Healthy adults (18–50 y) with BMIs of 25–31 kg/m^2^	17	14	N/A	Casein meal: subjects consumed a test drink containing casein protein powder, bread, butter, and jam.		N/A
						Whey meal: subjects consumed a test drink containing whey protein powder, bread, butter, and jam.		
						Skim milk meal: subjects consumed a test drink containing skim milk, bread, butter, and jam.		
Luscombe et al. (2003) [47]	Randomized, parallel	Adults with hyperinsulinemia and BMIs of 27–43 kg/m^2^	36	3	112	Higher-protein meal/diet: subjects followed a higher-protein hypocaloric diet for 12 weeks followed by a higher-protein weight maintenance diet for 4 weeks. Acute meals reflected the chronic diet and were consumed at weeks 0 and 16 for the measurement of DIT.	Lower-protein meal/diet: subjects followed a lower-protein hypocaloric diet for 12 weeks followed by a lower-protein weight maintenance diet for 4 weeks. Acute meals reflected the chronic diet and were consumed at weeks 0 and 16.	22
Luscombe-Marsh et al. (2005) [74]	Randomized, parallel	Adults (20–65 y) with hyperinsulinemia and BMIs of 27–40 kg/m^2^	30	3	112	Higher-protein meal/diet: subjects followed a higher-protein, low-fat hypocaloric diet for 12 weeks followed by a weight maintenance diet for 4 weeks. Acute meals reflected the chronic diet and were consumed at weeks 0 and 16 for the measurement of DIT.	Lower-protein meal/diet: subjects followed a standard-protein, high-fat hypocaloric diet for 12 weeks followed by a weight maintenance diet for 4 weeks. Acute meals reflected the chronic diet and were consumed at weeks 0 and 16 for the measurement of DIT.	20
Martens et al. (2015) [32]	Randomized, parallel	Healthy adults with normal weights or slightly elevated adiposity	42	24	84	Protein meals/diet: subjects received dietary guidance and 2 protein shakes/d for 12 weeks. Subjects consumed meals that reflected their diet group inside a respiration chamber during weeks 1 and 12.	Carbohydrate meals/diet: subjects received dietary guidance and 2 carbohydrate shakes/d for 12 weeks. Subjects consumed meals that reflected their diet group inside a respiration chamber during weeks 1 and 12.	25
Mikkelsen et al. (2000) [28]	Randomized, crossover	Healthy young men with BMIs of 26–32 kg/m^2^	12	24	4	Pork diet: subjects consumed a higher-protein diet (mainly from pork) for 4 d. Meals were consumed inside a respiration chamber on day 4.	Carbohydrate diet: subjects consumed a higher-carbohydrate diet for 4 d. Meals were consumed inside a respiration chamber on day 4.	18
						Soy diet: subjects consumed a higher-protein diet (mainly from soy) for 4 d. Meals were consumed inside a respiration chamber on day 4.		17
Neumann et al. (2016)^1^ [26]	Randomized, parallel	Healthy women (11–36 y); habitual breakfast skippers	16	2	6	Protein meal: subjects consumed a proprietary breakfast sandwich and Greek yogurt for breakfast for 8 d. Metabolism was measured on days 1 and 8.	Carbohydrate meal: subjects consumed an English muffin, yogurt, and cream cheese for breakfast for 8 d. Metabolism was measured on days 1 and 8.	23
Nguo et al. (2019) [30]	Randomized, crossover	Healthy adolescents (11–19 y) with normal weights or obesity	26	4	N/A	Protein meal: subjects consumed an acute meal comprising whey protein isolate, milk, vanilla ice cream, and canola oil.	Carbohydrate meal: subjects consumed an acute meal comprising maltodextrin, milk, vanilla ice cream, and canola oil.	50
Nielsen et al. (2018) [48]	Randomized, crossover	Healthy adults (18–50 y) with BMIs of 25–30 kg/m^2^	21	3	N/A	Cod meal: subjects consumed an acute meal containing cod and mashed potatoes or pasta.		N/A
						Veal meal: subjects consumed an acute meal containing veal and mashed potatoes or pasta.		
Nielsen et al. (2019) [49]	Randomized, crossover	Healthy adults (18–50 y) with BMIs of 25–30 kg/m^2^	25	3	N/A	Salmon meal: subjects consumed an acute meal containing salmon and mashed potatoes or pasta.		N/A
						Veal meal: subjects consumed an acute meal containing veal and mashed potatoes or pasta.		
Oliveira et al. (2021) [52]	Randomized, crossover	Healthy adults (18–35 y) with normal weights	43	32	N/A	Protein meal: subjects consumed soy protein nutritional supplements mixed with olive oil and low-fat milk or apple juice instead of standard meals.	Control meal: subjects consumed balanced meals comprising standard food items.	25
Ooi et al. (2021) [58]	Randomized, parallel	Healthy adults (21–45 y) with BMIs of 25–36 kg/m^2^	109	3	112	Higher-protein diet/meal + placebo diet: subjects consumed a higher-protein hypocaloric diet with placebo supplementation for 16 weeks. At baseline and 16 weeks, subjects consumed an acute meal that was representative of the chronic diet for the measurement of DIT.	Standard-protein diet/meal + BCAA: Subjects consumed a standard-protein hypocaloric diet with BCAA supplementation for 16 weeks. At baseline and 16 weeks, subjects consumed an acute meal that was representative of the chronic diet for the measurement of DIT.	13
							Standard-protein diet/meal + placebo: subjects consumed a standard-protein hypocaloric diet with BCAA supplementation for 16 weeks. At baseline and 16 weeks, subjects consumed an acute meal that was representative of the chronic diet for the measurement of DIT.	13
Raben et al. (2003)^1^ [43]	Randomized, crossover	Healthy adults (20–30 y) with BMIs >18.5 and <25 kg/m^2^	19	5	N/A	Protein meal: subjects consumed an acute meal comprising bread, cheese, yogurt, muesli, egg, and skim milk.	Carbohydrate meal: subjects consumed an acute meal comprising corn flakes, skim milk, white bread, butter, cheese, jam, and honey.	20
							Fat meal: subjects consumed an acute meal comprising yogurt mixed with cream, apple, honeydew, rye bread, butter, cream cheese, and whole milk.	20
Riggs et al. (2007) [65]	Randomized, crossover	Healthy women (19–28 y) with BMI classifications of underweight, normal weight, or overweight	21	3.5	N/A	Higher-protein meal: subjects consumed 2 Atkins Advantage bars.	Lower-protein meal: subjects consumed 2 OmegaZone bars.	6
Sambashivaiah et al. (2023) [77]	Randomized, crossover	Healthy adult males (20–35 y)	15	5	N/A	Higher-protein meals: subjects consumed liquid meals containing 30% whey protein and 30% soy protein on 2 separate days.	Lower-protein meals: subjects consumed liquid meals containing 15% whey protein and 15% soy protein on 2 separate days.	15
Schutz et al. (1987)^1^ [33]	Nonrandomized, crossover	Healthy elderly men (65–74 y) with normal weights	6	5	42	Higher-protein meal/diet: subjects consumed a habitual protein diet for 6 weeks. During week 3, a test meal was provided that contained liquid semipurified formula that reflected the chronic diet for the measurement of DIT.	Lower-protein meal/diet: subjects consumed a diet containing protein at the level of physiological requirement for 6 weeks. During week 3, a test meal was provided that contained liquid semipurified formula that reflected the chronic diet for the measurement of DIT.	9
Scott et al. (2005)^1^ [60]	Randomized, crossover	Healthy adults with normal weights	8	3	N/A	High-protein meal: subjects consumed a high-protein, low-calorie liquid meal replacement shake.	Lower-protein meal: subjects consumed a lower-protein, low-calorie liquid meal-replacement shake.	45
Smeets et al. (2008) [45]	Randomized, crossover	Healthy adults (18–60 y) with BMIs of 20–30 kg/m^2^	30	3.5	N/A	Higher-protein meal: subjects consumed an acute meal comprising pasta, sausage (higher level of protein), and tomato sauce.	Lower-protein meal: subjects consumed an acute meal comprising pasta, sausage (adequate level of protein), and tomato sauce.	15
Smeets et al. (2013) [61]	Randomized, crossover	Healthy adults	24	24	N/A	Higher-protein meals: subjects consumed higher-protein meals that provided 100% or 80% of energy needs inside a respiration chamber.	Lower-protein meals: subjects consumed lower-protein meals that provided 100% or 80% of energy needs inside a respiration chamber.	15
Stiegler et al. (2008)^1^ [35]	Randomized, parallel	Healthy adults with normal weights or obesity	20	4	N/A	Protein meal: subjects consumed an acute meal comprising milk, Pure Soya Protein Isolate Powder, flavored nutritional drink (Complan), and long-chain TG fat emulsion (Calogen).	Carbohydrate meal: subjects consumed an acute meal comprising milk, powdered glucose polymer, flavored nutritional drink (Complan), and long-chain TG fat emulsion (Calogen).	33
Suen et al. (2003) [40]	Randomized, crossover	Healthy women (20–45 y) with BMIs of 32–59 kg/m^2^	7	6	7	Protein meal/diet: subjects consumed a hypocaloric diet that contained 43% of energy from protein. On day 7, DIT was measured after the consumption of an acute meal that reflected the macronutrient distribution of the chronic diet.	Carbohydrate meal/diet: subjects consumed a hypocaloric diet that contained 72 of energy from carbohydrate. On day 7, DIT was measured after the consumption of an acute meal that reflected the macronutrient distribution of the chronic diet.	31
							Fat meal/diet: subjects consumed a hypocaloric diet that contained 68% of energy from fat. On day 7, DIT was measured after the consumption of an acute meal that reflected the macronutrient distribution of the chronic diet.	21
Surowska et al. (2019) [64]	Randomized, crossover	Healthy sedentary adults	12	7.5	6	Higher-protein diet: subjects consumed a 3-d weight maintenance diet followed by a 6-d higher-protein hypercaloric diet. Overfeeding was achieved by adding 6 drinks/d containing skim milk and sucrose. DIT was measured on days 0 and 7 in response to meals that reflected the weight maintenance and hypercaloric diets, respectively.	Lower-protein diet: subjects consumed a 3-d weight maintenance diet followed by a 6-d low-protein hypercaloric diet. Overfeeding was achieved by adding 6 drinks/d containing water, lactose, and sucrose. DIT was measured on days 0 and 7 in response to meals that reflected the weight maintenance and hypercaloric diets, respectively.	15
Tan et al. (2010) [50]	Randomized, crossover	Healthy adults (≥18 y) with normal weights or overweight	12	8	N/A	Meat meal: subjects consumed an acute meal containing ham, steak, toast, tomato, fruit juice, potatoes, and vegetables.		N/A
						Dairy meal: subjects consumed an acute meal containing a chocolate milk shake, cheese, yogurt, toast, margarine, jam, and salad.		
						Soy meal: subjects consumed an acute meal containing a chocolate soy shake, soy powder, soy cheese, soy yogurt, toast, butter, jam, and salad.		
Veldhorst et al. (2010) [59]	Randomized, crossover	Healthy adults (18–40 y) with BMIs of 18.5–25 kg/m^2^	22	24	N/A	Higher-protein meals: subjects consumed meals containing a chicken filet, milk, ham, tuna, soya milk, feta cheese, yogurt, bread, vegetables, soup, rice, muesli, and sugar-free syrup.	Lower-protein meals: subjects consumed meals containing chocolate spread, confiture, coffee or tea, bread, low-fat margarine, cheese, vegetables, olive oil, grape juice, soup, noodles, and fruit cocktail.	20
Verboeket-van de Venn et al. (1996) [66]	Randomized, parallel	Healthy adults (19–35 y) with BMIs of 21–28 kg/m^2^	16	36	183	Higher-protein diet: subjects consumed a higher-protein diet comprised of reduced-fat products. At baseline, 3, and 6 mo, meals were consumed inside a respiration chamber.	Lower-protein diet: subjects consumed a lower-protein diet comprising full-fat products. At baseline, 3, and 6 mo, meals were consumed inside a respiration chamber.	6
Walsh et al. (2013) [44]	Randomized, crossover	Healthy adults (18–40 y) with BMIs ≥27 kg/m^2^	8	5	28	Protein meal/diet: after 10%–15% weight loss in the run-in phase, subjects consumed a higher-protein, low-carbohydrate diet for 4 weeks. At the end of the 4-week period, DIT was measured after consumption of an acute meal that reflected the chronic diet (egg, sausage, and cheese bake).	Low GI meal/diet: after 10%–15% weight loss in the run-in phase, subjects consumed a low GI diet for 4 weeks. At the end of the 4-week period, DIT was measured after consumption of an acute meal that reflected the chronic diet (steel cut oats, egg, cottage cheese, margarine, grapefruit, fructose sweetener).	10
							Low-fat (high GI) meal/diet: after 10%–15% weight loss in the run-in phase, subjects consumed a low-fat, high GI diet for 4 weeks. At the end of the 4-week period, DIT was measured after consumption of an acute meal that reflected the chronic diet (instant oatmeal, turkey sausage, margarine, nonfat milk, grape juice, raisins, sugar).	10
Westerterp et al. (1999) [76]	Randomized, crossover	Healthy women (23–33 y) with normal weights	8	36	N/A	Protein meals: subjects consumed higher-protein meals and snacks that comprised “normal food items” during a 36-h stay in a respiration chamber.	Fat meals: subjects consumed high-fat meals and snacks that comprised “normal food items” during a 36-h stay in a respiration chamber.	20
Westerterp-Plantenga et al. (2009) [57]	Randomized, crossover	Healthy males (18–40 y) with BMIs of 20–25 kg/m^2^	10	36	N/A	Higher-protein meals: subjects consumed meals containing chicken, soy milk, tuna, ham, yogurt, milk, merengue, feta cheese, rice, bread, butter, vegetables, soup, and muesli.	Lower-protein meals: subjects consumed meals containing chicken, soy dessert, tuna, cottage cheese, bread, orange juice, fruits, vegetables, butter, soup, and muesli.	20
Whitehead et al. (1996) [37]	Randomized, crossover	Healthy adults with overweight or obesity	8	24	7	Protein diet: subjects consumed a diet that contained 36% of energy from protein for 7 d. During the last day, meals were consumed inside a calorimeter chamber.	Carbohydrate diet: subjects consumed a diet that contained 53% of energy from carbohydrate for 7 d. During the last day, meals were consumed inside a calorimeter chamber.	21
							Fat diet: subjects consumed a diet that contained 53% of energy from fat for 7 d. During the last day, meals were consumed inside a calorimeter chamber.	21
Xiong et al. (2022)^1^ [34]	Randomized, crossover	Healthy men (18–45 y) with normal weights or overweight/obesity	39	3	N/A	Protein meal: subjects consumed an acute meal comprising chicken, beef, shrimp, tofu, rice, vegetables, and potatoes.	Carbohydrate meal: subjects consumed an acute meal comprising rice, Garland Chrysanthemum, fruit, vegetables, eggs, red bean, lyceum Chinese, and sugar.	29
							Fat meal: subjects consumed an acute meal comprising streaky pork, beancurd sheets, fish meat, vegetables, tomatoes, and rice.	20

Abbreviations: %BF, percent body fat; BCAA, branched chain amino acid; DIT, diet-induced thermogenesis; DLW, doubly labeled water; GI, glycemic index; NCEP, National Cholesterol Education Program; TDEE, total daily energy expenditure; TG, triglyceride.

1 Data were not extracted from 1 or more treatment conditions within the study because the treatment condition was exclusionary (e.g., not a mixed meal/diet, not isocaloric with other treatment conditions, contained alcohol, involved exercise, included pregnant women, irrelevant to analysis).

2 The duration of the metabolism measurement (h) was recorded as N/A because doubly labeled water samples were collected for 14 d to estimate energy expenditure.

3 Leidy et al. [70] used an acute on chronic study design in which subjects consumed a higher-protein or lower-protein hypocaloric diet for 12 wk. During week 9, all subjects consumed a higher-protein and a normal-protein meal on 2 separate days for the measurement of DIT. Data for the effect of the acute meals during week 9 were extracted.

Thirty-three studies were acute meal challenges, 13 were chronic studies, and 6 measured both the acute and chronic effects. Eleven of the studies involved hypocaloric meals/diets, 2 studies involved hypercaloric meals/diets, and 2 studies involved a weight loss phase followed by a weight maintenance phase. The chronic studies ranged from 4 d [28] to 1 y [75]. Thirty-seven studies investigated the effect of the amount of protein on energy metabolism, 7 investigated the effect of the type of protein, and 8 evaluated both. Fifty studies involved healthy individuals with or without overweight/obesity, with the remaining 2 studies in subjects with hyperinsulinemia. There was a wide variety of intervention and comparator meals/diets. For trials that studied different amounts of protein, comparator diets replaced protein with carbohydrate, fat, or a combination of the 2. The difference in the percentage of total energy intake from protein between interventions and comparators ranged from 6% [66] to 60% [29]. Additionally, the trials that compared types of protein involved whey, casein, soy, gelatin, pork, salmon, veal, cod, dairy, mycoprotein, and/or chicken.

### Meta-analysis of acute meals containing different amounts of protein

#### DIT and TDEE

**FIGURE 2:**
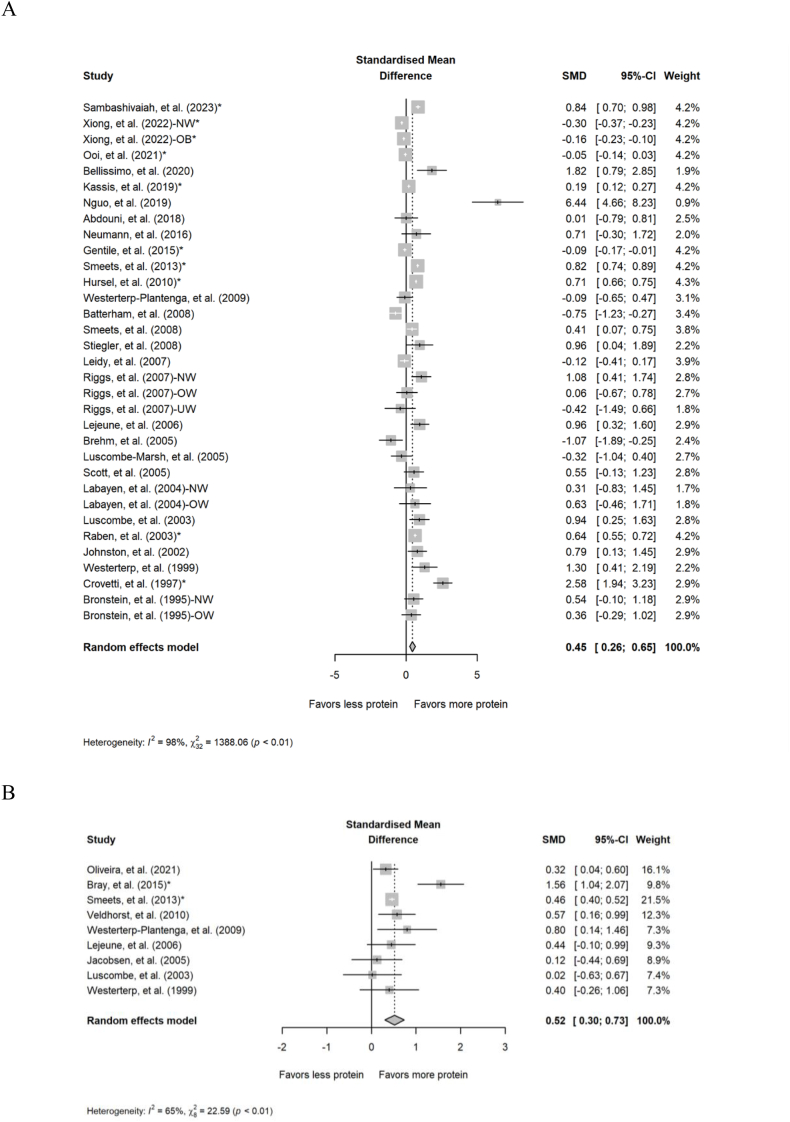
Forest plot of the meta-analysis on the effect of acute meals and diets containing different amounts of protein on (A) diet-induced thermogenesis and (B) total daily energy expenditure. CI, confidence interval; NW, normal weight; OB, obese; SMD, standardized mean difference; UW, underweight. ∗Effect sizes for correlated comparisons within a study were pooled before running final analyses.

Overall, 33 comparisons reported from 28 studies were included in the analysis of the impact of acute meals containing different amounts of protein on DIT that was measured for a range of 2–36 h. Intake of higher-protein meals resulted in higher DIT compared with lower-protein meals (SMD: 0.45; 95% CI: 0.26, 0.65; *P* < 0.001; Figure 2A) but the data displayed substantial heterogeneity (*Q* = 1388; *P* < 0.001; *I*^2^ = 97.7%). For TDEE, 9 comparisons reported from 9 studies were included in the analysis of the impact of acute diets (24–48 h) containing different amounts of protein. Intake of acute diets containing higher protein increased TDEE compared with diets containing lower amounts of protein (SMD: 0.52; 95% CI: 0.30, 0.73; *P* < 0.001; Figure 2B) but the data displayed substantial heterogeneity (*Q* = 22.6; *P =* 0.004; *I*^2^ = 64.6%).

Findings from the subgroup analyses of DIT and TDEE in acute studies are presented in Table 2. The effect of higher protein compared with lower-protein meals on DIT remained significant when the difference in percent energy from protein between groups was ≥19.6% but not <19.6%. Additionally, higher-protein meals resulted in significantly greater DIT for the subgroups with BMI below the median (<23.8 kg/m^2^), age below the median (<26.1 y), and low risk of bias but not the subgroups involving BMIs and age equal to or above the median or high risk of bias/some concerns. An additional weight status subgroup separated studies based on whether the authors reported that they recruited participants with underweight, normal weight, overweight, obesity, or a combination of these categories. Because the results for BMI and weight status subgroups were not materially different, only the results for the BMI median split subgroups are reported throughout the manuscript. For TDEE, the effect of higher compared with lower-protein meals on TDEE remained significant for all subgroups of protein difference, sex, BMI, age, and blinding.

**TABLE 2:** Subgroup analyses of the acute effect of higher compared with lower-protein meals on DIT and TDEE.

Outcome and subgroups	*n*	SMD (95% CI); *P* value	Q; *P* value	*I*^2^ (%)
Diet-induced thermogenesis				
Protein difference (median split)^1^				
<19.6%	15	0.296 (0.00, 0.593); *P* = 0.051	424; *P* < 0.001	96.7
≥19.6%	18	0.601 (0.321, 0.881); *P* < 0.001	963; *P* < 0.001	98.2
% Male (median split)				
<38.5%	19	0.450 (0.138, 0.762); *P* = 0.005	137; *P* < 0.001	86.9
≥38.5%	14	0.462 (0.179, 0.746); *P* = 0.001	1184; *P* < 0.001	98.9
BMI (kg/m^2^) (median split)				
<23.8	16	0.693 (0.379, 1.01); *P* < 0.001	684; *P* < 0.001	97.8
≥23.8	17	0.222 (–0.024, 0.468); *P* = 0.077	531; *P* < 0.001	97.0
Age (y) (median split)				
<26.1	15	0.796 (0.489, 1.10); *P* < 0.001	571; *P* < 0.001	97.5
≥26.1	16	0.172 (–0.080, 0.423); *P* = 0.181	568; *P* < 0.001	97.4
Study design				
Parallel	8	0.314 (–0.062, 0.690); *P* = 0.102	16.8; *P* = 0.019	58.2
Crossover	25	0.482 (0.261, 0.704); *P* < 0.001	1304; *P* < 0.001	98.2
Blinding				
Single/double	20	0.389 (0.143, 0.634); *P* = 0.002	1232; *P* < 0.001	98.5
Open-label	13	0.577 (0.188, 0.967); *P* = 0.004	101; *P* < 0.001	88.1
Number of meals consumed during metabolism measurement				
>1	5	0.418 (–0.285, 1.12); *P* = 0.244	49.9; *P* < 0.001	92.0
1	28	0.458 (0.250, 0.666); *P* < 0.001	1158; *P* < 0.001	97.7
Risk of bias				
Low	26	0.526 (0.299, 0.754); *P* < 0.001	1111; *P* < 0.001	97.8
High/some concerns	7	0.221 (–0.242, 0.683); *P* = 0.350	257; *P* < 0.001	97.7
Total daily energy expenditure				
Protein difference (median split)^1^				
<20%	3	0.456 (0.396, 0.517); *P* < 0.001	1.78; *P* = 0.410	0.00
≥20%	7	0.512 (0.215, 0.808); *P* < 0.001	12.3; *P* = 0.057	51.0
% Male (median split)				
<50%	5	0.365 (0.122, 0.607); *P* = 0.003	2.81; *P* = 0.590	0.00
≥50%	4	0.708 (0.320, 1.10); *P* < 0.001	19.1; *P* < 0.001	84.3
BMI (kg/m^2^) (median split)				
<25	5	0.439 (0.246, 0.633); *P* < 0.001	2.24; *P* = 0.692	0.00
≥25	4	0.553 (0.008, 1.10); *P* = 0.047	20.3; *P* < 0.001	85.2
Age (y) (median split)				
<24.85	4	0.371 (0.172, 0.570); *P* < 0.001	1.83; *P* = 0.609	0.00
≥24.85	5	0.654 (0.197, 1.11); *P* = 0.005	19.9; *P* < 0.001	79.9
Study design				
Parallel	—	—	—	—
Crossover	7	0.454 (0.396, 0.511); *P* < 0.001	3.60; *P* = 0.731	0.00
Blinding				
Single/double	5	0.731 (0.361, 1.10); *P* < 0.001	18.2; *P* = 0.001	78.0
Open-label	4	0.266 (0.046, 0.486); *P* = 0.018	1.09; *P* = 0.779	0.00
Risk of bias				
Low	8	0.533 (0.214, 0.853); *P* = 0.001	22.4; *P* = 0.002	68.7
High/some concerns	—	—	—	—

Abbreviations: DIT, diet-induced thermogenesis; SMD, standardized mean difference; TDEE, total daily energy expenditure.

Individual effect sizes within each subgroup were pooled using random-effects models that utilized inverse-variance weighting and the DerSimonian–Laird estimator. Correlated, within-study comparisons that fell in the same subgroup were pooled before running each subgroup analysis. Heterogeneity within subgroups was assessed using Cochran’s *Q* and the *I*^2^ statistic (low heterogeneity: 0%–40%; moderate/higher heterogeneity: >40%). Analyses were not performed for subgroups with fewer than 3 comparisons after addressing multiple within-study comparisons.

1 The protein difference represents the difference in percent of energy from protein in the higher and lower-protein groups.

#### Exploratory subgroup and sensitivity analyses for DIT in acute studies

To further evaluate how BMI impacts acute DIT responses to higher compared with lower-protein meals, this subgroup analysis was conducted in crossover studies only. The effect of higher compared with lower-protein meals on DIT remained significant for the subgroup with BMI below the median of 23.4 kg/m^2^ (SMD: 0.76; 95% CI: 0.41, 1.12; *P* < 0.001; *N* = 12) but not the subgroup above the median (SMD: 0.24; 95% CI: –0.06, 0.53; *P* > 0.05; *N* = 13).

In addition, sensitivity analyses were conducted in which similar studies were grouped together based on BMI category, protein difference, duration of DIT measurement, and meal size (Supplemental Table 1). In alignment with the original subgroup analyses, the 7 subgroup analyses involving participants with BMIs in the normal weight category indicated that higher-protein intake resulted in significantly higher DIT response, whereas there was no significant effect in the 6 analyses involving participants with BMIs in the overweight or obesity categories. However, there were 2 subgroup analyses involving participants with overweight/obesity that were borderline statistically significant (*P* ≤ 0.08). First, the subgroup analysis that included 3 studies in participants with overweight/obesity, protein differences of 20%–33%, and a meal containing <550 kcal approached significance (SMD: 0.44; 95% CI: –0.06, 0.93; *P =* 0.084). The measurement duration for these studies ranged from 2 to 5.5 h. Conversely, the subgroup analysis that included 2 studies in participants with overweight/obesity, protein differences of 20%–33%, and a meal containing ≥550 kcal did not show a significant effect (SMD: 0.32; 95% CI: –0.92, 1.55; *P =* 0.617). Additionally, the subgroup analysis that included 3 studies in participants with overweight/obesity, protein differences of 20%–33%, and a measurement duration of ≥4 h approached significance (SMD: 0.59; 95% CI: –0.02, 1.2; *P =* 0.058). In comparison, the subgroup analysis that included 2 studies involving participants with overweight/obesity, protein differences of 20%–33%, and a measurement duration of <4 h did not approach significance (SMD: 0.13; 95% CI: –0.87, 1.1; *P =* 0.794). To further explore how measurement duration impacted the divergent results for DIT in participants with and without obesity, the measurement duration was graphed against the SMD for the 18 effects that were included when similar studies were grouped together (excluding the studies that involved long measurements in metabolic chambers) (Supplemental Figure 1). Notably, the SMD was ≥0 for all effects involving participants with normal weight (Supplemental Figure 1B), but there was more variability in SMDs for the effects involving participants with overweight/obesity (4 were <0 and 5 were ≥0) (Supplemental Figure 1C). Furthermore, for the effects from studies that measured DIT for ≥4 h in participants with overweight/obesity, 4 SMDs were ≥0 and 1 SMD was <0.

#### Postprandial substrate utilization

**FIGURE 3:**
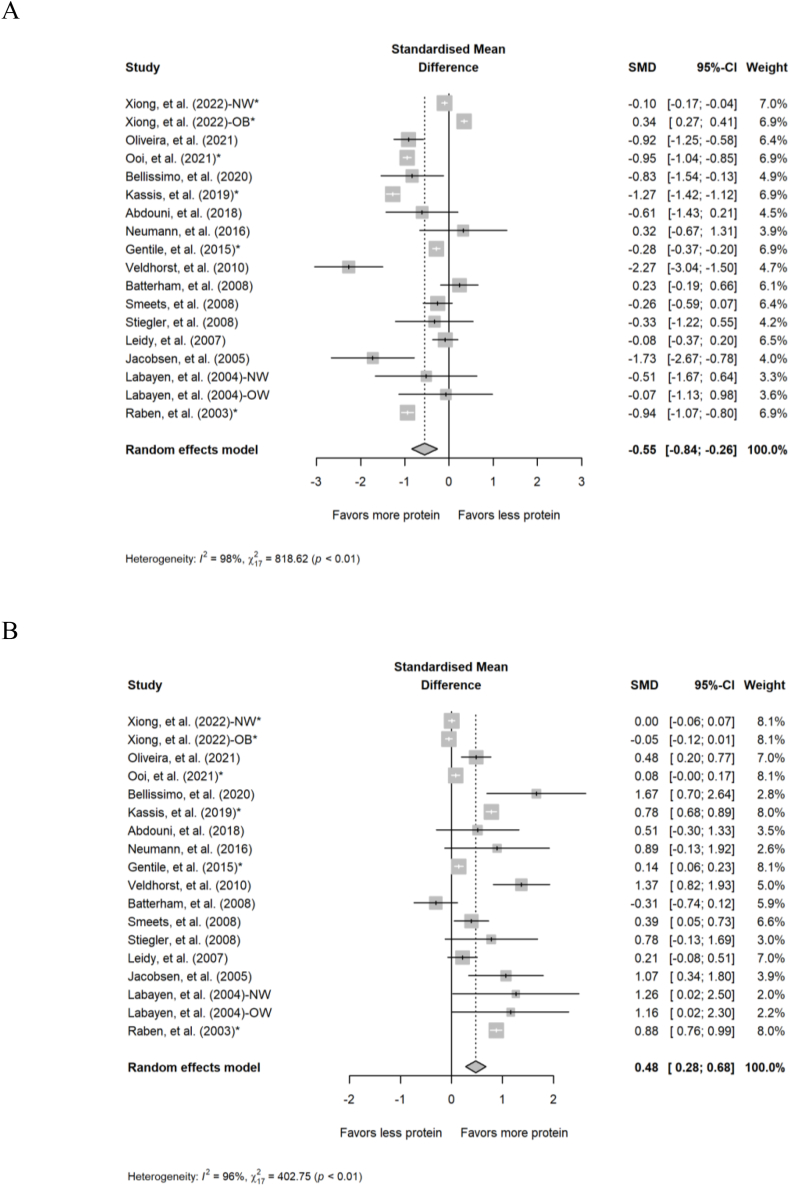
Forest plot of the effect of acute diets with different amounts of protein on postprandial (A) carbohydrate oxidation and (B) fat oxidation. CI, confidence interval; NW, normal weight; OB, obese; SMD, standardized mean difference. ∗Effect sizes for correlated comparisons within a study were pooled before running final analyses.

**FIGURE 4:**
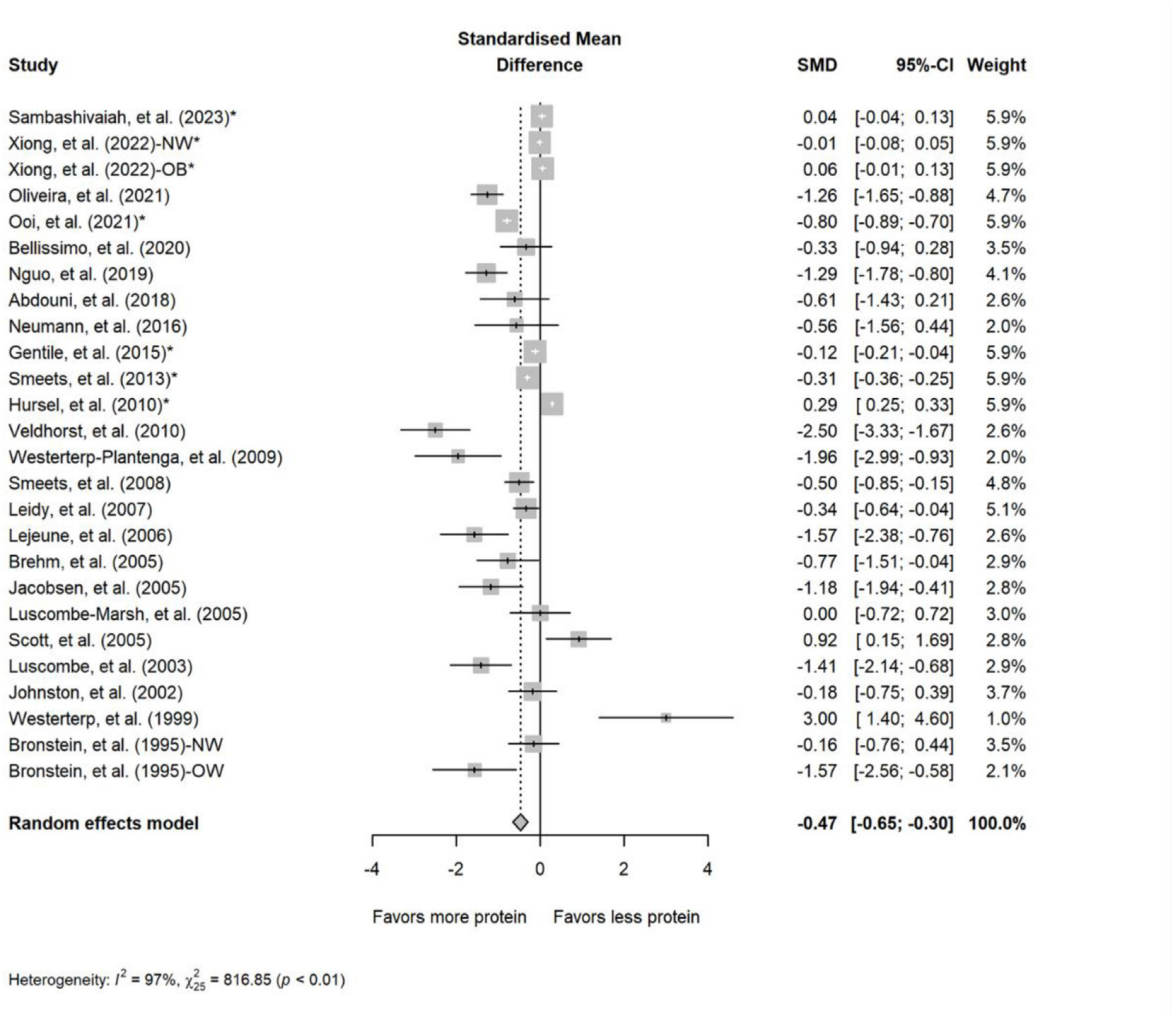
Forest plot of the effect of acute diets with different amounts of protein on postprandial respiratory exchange ratio. CI, confidence interval; NW, normal weight; OB, obese; SMD, standardized mean difference. ∗Effect sizes for correlated comparisons within a study were pooled before running final analyses.

Overall, 18 comparisons from 16 studies were included in the analyses of acute intake of different amounts of protein on postprandial carbohydrate and fat oxidation. Diets containing higher protein reduced postprandial carbohydrate oxidation (SMD: –0.55; 95% CI: –0.84, –0.26; *P* < 0.001; Figure 3A) and increased postprandial fat oxidation (SMD: 0.48; 95% CI: 0.28, 0.68; *P* < 0.001; Figure 3B) compared with diets with lower protein. The data displayed substantial heterogeneity for both postprandial carbohydrate oxidation (*Q* = 819; *P* < 0.001; *I*^2^ = 97.9%) and fat oxidation (*Q* = 403; *P* < 0.001; *I*^2^ = 95.8%). Finally, for postprandial RER, 26 comparisons from 24 studies were included in the analysis of acute intake of different amounts of protein. Diets containing higher protein reduced the RER (indicating a greater ratio of fat to carbohydrate oxidation) compared with diets with lower protein (SMD: –0.47; 95% CI: –0.65, –0.30; *P* < 0.001; Figure 4) but the data displayed substantial heterogeneity (*Q* = 817; *P* < 0.001; *I*^2^ = 96.9%). Findings from the subgroup analyses of postprandial substrate utilization in acute studies are presented in Supplemental Table 2.

### Meta-analysis of chronic diets containing different amounts of protein

#### DIT, TDEE, and REE

**FIGURE 5:**
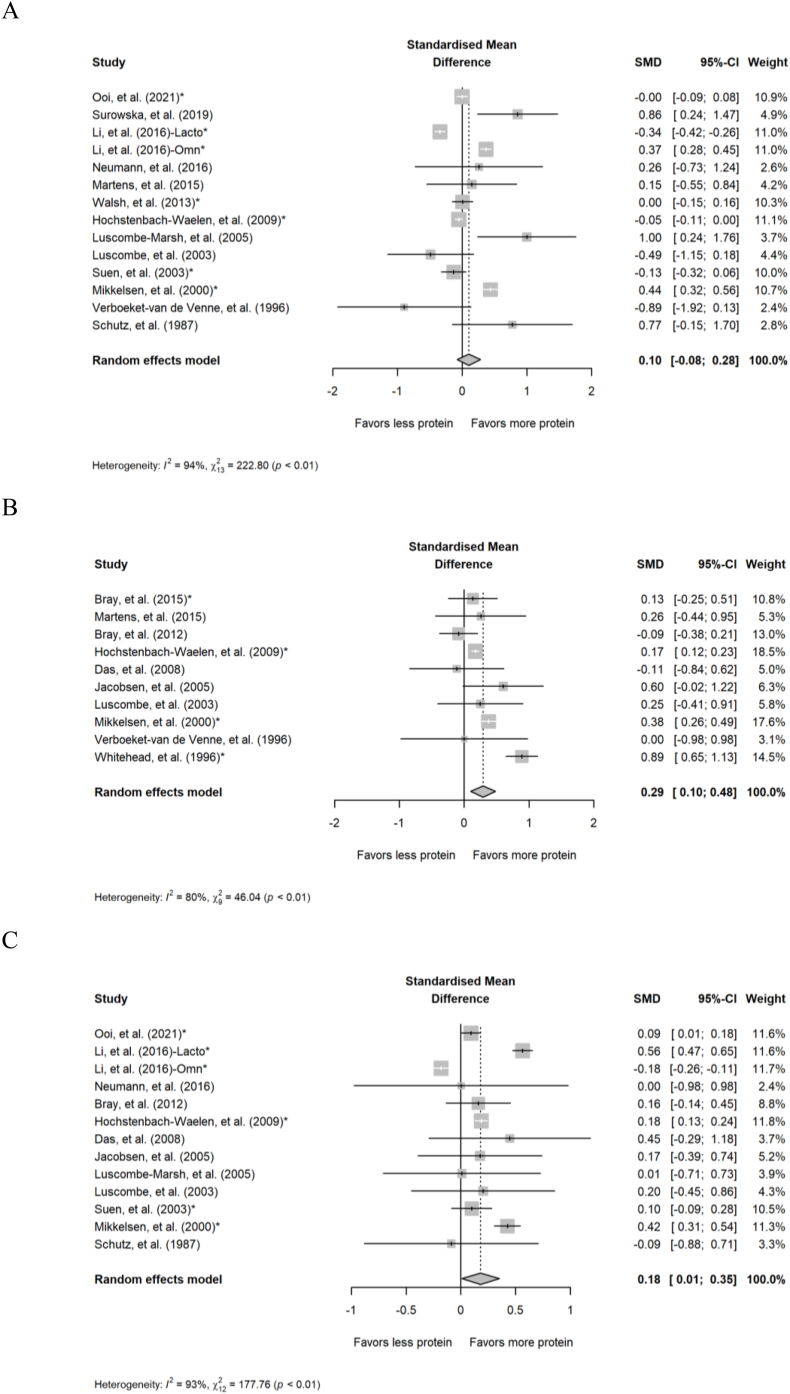
Forest plot of the meta-analysis on the effect of chronic diets containing different amounts of protein on (A) diet-induced thermogenesis (DIT), (B) total daily energy expenditure, and (C) resting energy expenditure. CI, confidence interval; lacto, lacto-ovo vegetarian; omn, omnivorous; RMR, resting metabolic rate; SMD, standardized mean difference. ∗Effect sizes for correlated comparisons within a study were pooled before running final analyses.

The 14 comparisons from 13 studies demonstrated no statistically significant impact of diets containing higher compared with lower protein on DIT (SMD: 0.10; 95% CI: –0.08, 0.28; *P =* 0.27; Figure 5A) but the data displayed substantial heterogeneity (*Q* = 223; *P* < 0.001; *I*^2^ = 94.2%). There were 10 comparisons reported in 10 studies for TDEE, and 13 comparisons from 12 studies for REE. Chronic diets containing higher protein increased TDEE (SMD: 0.29; 95% CI: 0.10, 0.48; *P =* 0.003; Figure 5B) and REE (SMD: 0.18; 95% CI: 0.01, 0.35; *P =* 0.039; Figure 5C) compared with diets containing lower amounts of protein. There was substantial heterogeneity for the TDEE (*Q* = 46.0; *P* < 0.001; *I*^2^ = 80.5%) and REE analyses (*Q* = 178; *P* < 0.001; *I*^2^ = 93.2%).

Findings from the subgroup analyses of energy expenditure in chronic studies are presented in Table 3. In alignment with the main analysis, there was no significant impact of chronic diets containing higher compared with lower protein on DIT for any subgroup. For TDEE, the effect of higher protein compared with lower-protein diets was significant for both subgroups of protein difference, although the effect was more pronounced for protein differences ≥20% than protein differences <20%. In addition, the effect was significant for the subgroups with younger mean ages (<26 y) but not older mean ages (≥26 y). Furthermore, the effect was significant for the subgroups with eucaloric dietary interventions, shorter intervention lengths (<31.5 d), and low risk of bias, but not for the subgroups with hypocaloric dietary interventions, longer intervention lengths (≥31.5 d), and high risk of bias/some concerns.

**TABLE 3:** Subgroup analyses of the chronic effect of higher compared with lower-protein diets on energy expenditure.

Outcome and subgroups	*N*	SMD (95% CI); *P* value	Q; *P* value	*I*^2^ (%)
Diet-induced thermogenesis				
Protein difference (median split)^1^				
<15%	6	0.025 (–0.148, 0.197); *P* = 0.777	10.5; *P* = 0.063	52.3
≥15%	8	0.197 (–0.076, 0.470); *P =* 0.157	70.3; *P* < 0.001	90.0
% Male (median split)				
<48%	7	0.069 (–0.313, 0.451); *P =* 0.725	157; *P* < 0.001	96.2
≥48%	7	0.143 (–0.054, 0.339); *P =* 0.154	64.7; *P* < 0.001	90.7
BMI (kg/m^2^) (median split)				
<29.55	7	0.250 (–0.091, 0.592); *P =* 0.151	64.4; *P* < 0.001	90.7
≥29.55	7	0.013 (–0.250, 0.277); *P =* 0.920	157; *P* < 0.001	96.2
Age (y) (median split)				
<31.55	7	0.161 (–0.109, 0.431); *P* = 0.243	62.1; *P* < 0.001	90.3
≥31.55	7	0.069 (–0.228, 0.365); *P =* 0.650	160; *P* < 0.001	96.2
Study design				
Parallel	6	0.024 (–0.362, 0.411); *P =* 0.901	12.0; *P =* 0.034	58.5
Crossover	8	0.141 (–0.094, 0.376); *P =* 0.241	211; *P* < 0.001	96.7
Blinding				
Single/double	6	0.077 (–0.116, 0.269); *P =* 0.436	54.9; *P* < 0.001	90.9
Open label	8	0.147 (–0.215, 0.510); *P =* 0.425	168; *P* < 0.001	95.8
Energy balance				
Hypocaloric	5	–0.079 (–0.395, 0.237); *P =* 0.624	150; *P* < 0.001	97.3
Eucaloric	7	0.190 (–0.086, 0.466); *P =* 0.178	63.7; *P* < 0.001	90.6
Hypercaloric	—	—	—	—
Intervention length (d) (median split)				
<28	5	0.202 (–0.110, 0.514); *P =* 0.205	61.6; *P* < 0.001	93.5
≥28	9	0.047 (–0.223, 0.317); *P =* 0.734	161; *P* < 0.001	95.0
Risk of bias				
Low	8	0.195 (–0.080, 0.470); *P =* 0.164	214; *P* < 0.001	96.7
High/some concerns^2^	6	–0.025 (–0.140, 0.090); *P =* 0.669	7.44; *P =* 0.190	32.8
Total daily energy expenditure				
Protein difference (median split)^1^				
<20%	7	0.201 (0.041, 0.361); *P =* 0.014	16.1; *P =* 0.014	62.6
≥20%	4	0.498 (0.053, 0.943); *P =* 0.028	6.86; *P =* 0.077	56.2
% Male (median split)				
<48%	5	0.453 (0.052, 0.854); *P =* 0.027	10.6; *P =* 0.032	62.2
≥48%	5	0.186 (0.028, 0.345); *P =* 0.021	13.8; *P =* 0.008	71.1
BMI (kg/m^2^) (median split)				
<26.975	5	0.175 (0.119, 0.230); *P* < 0.001	2.04; *P =* 0.728	0.00
≥26.975	5	0.314 (–0.044, 0.672); *P =* 0.086	28.6; *P* < 0.001	86.0
Age (y) (median split)				
<26	4	0.175 (0.119, 0.231); *P* < 0.001	1.92; *P =* 0.589	0.00
≥26	6	0.290 (–0.047, 0.627); *P =* 0.092	29.2; *P* < 0.001	82.9
Study design				
Parallel	6	0.032 (–0.165, 0.229); *P =* 0.751	1.87; *P =* 0.867	0.00
Crossover	4	0.472 (0.194, 0.751); *P* < 0.001	40.0; *P* < 0.001	92.5
Blinding				
Single/double	6	0.184 (0.034, 0.333); *P =* 0.016	14.4; *P =* 0.013	65.3
Open label	4	0.576 (0.179, 0.972); *P =* 0.004	5.99; *P =* 0.112	49.9
Energy balance				
Hypocaloric	4	0.262 (–0.357, 0.880); *P =* 0.407	28.2; *P* < 0.001	89.3
Eucaloric	5	0.280 (0.113, 0.447); *P =* 0.001	11.4; *P =* 0.022	65.0
Hypercaloric	—	—	—	—
Intervention length (d) (median split)				
<31.5	4	0.472 (0.194, 0.751); *P* < 0.001	40.0; *P* < 0.001	92.5
≥31.5	6	0.032 (–0.165, 0.229); *P =* 0.751	1.87; *P =* 0.867	0.00
Risk of bias				
Low	7	0.366 (0.145, 0.587); *P =* 0.001	41.2; *P* < 0.001	85.4
High/some concerns	3	–0.032 (–0.293, 0.229); *P =* 0.811	0.790; *P =* 0.673	0.00
Resting energy expenditure				
Protein difference (median split)^1^				
<15%	7	0.102 (0.025, 0.179); *P =* 0.010	5.63; *P =* 0.466	0.00
≥15%	8	0.219 (0.069, 0.370); *P =* 0.004	21.2; *P =* 0.004	67.0
% Male (median split)				
<35%	6	0.026 (–0.187, 0.239); *P =* 0.808	12.2; *P =* 0.033	58.9
≥35%	7	0.263 (0.086, 0.440); *P =* 0.004	74.8; *P* < 0.001	92.0
BMI (kg/m^2^) (median split)				
<29.75	6	0.269 (0.093, 0.445); *P =* 0.003	13.8; *P =* 0.017	63.8
≥29.75	7	0.140 (–0.136, 0.415); *P =* 0.320	154; *P* < 0.001	96.1
Age (y) (median split)				
<35.71	6	0.226 (0.091, 0.361); *P =* 0.001	20.8; *P* < 0.001	75.9
≥35.71	7	0.126 (–0.232, 0.483); *P =* 0.492	154; *P* < 0.001	96.1
Study design				
Parallel	6	0.100 (0.021, 0.179); *P =* 0.013	1.24; *P =* 0.941	0.00
Crossover	7	0.196 (–0.047, 0.438); *P =* 0.114	173; *P* < 0.001	96.5
Blinding				
Single/double	7	0.200 (0.088, 0.312); *P* < 0.001	21.8; *P =* 0.001	72.4
Open label	6	0.129 (–0.322, 0.580); *P =* 0.575	153; *P* < 0.001	96.7
Energy balance				
Hypocaloric	7	0.174 (–0.102, 0.451); *P =* 0.216	155; *P* < 0.001	96.1
Eucaloric	5	0.251 (0.066, 0.437); *P =* 0.008	13.6; *P =* 0.009	70.6
Hypercaloric	—	—	—	—
Intervention length (d) (median split)				
<28	5	0.233 (0.078, 0.388); *P =* 0.003	14.7; *P =* 0.006	72.7
≥28	8	0.154 (–0.134, 0.443); *P =* 0.294	155; *P* < 0.001	95.5
Risk of bias^2^				
Low	9	0.223 (–0.020, 0.465); *P =* 0.072	173; *P* < 0.001	95.4
High/some concerns	4	0.095 (0.021, 0.169); *P =* 0.012	0.390; *P =* 0.941	0.00

Abbreviation: DIT, diet-induced thermogenesis.

Individual effect sizes within each subgroup were pooled using random-effects models that utilized inverse-variance weighting and the DerSimonian–Laird estimator. Correlated, within-study comparisons that fell in the same subgroup were pooled before running each subgroup analysis. Heterogeneity within subgroups was assessed using Cochran’s *Q* and the *I*^2^ statistic (low heterogeneity: 0%–40%; moderate/higher heterogeneity: >40%). Analyses were not performed for subgroups with fewer than 3 comparisons after addressing multiple within-study comparisons.

1 The protein difference represents the difference in percent of energy from protein in the higher and lower-protein groups.

2 The “high/some concerns” subgroup for risk of bias included the 1 nonrandomized study by Schutz et al. [33].

Similar to the main analysis, the effect of higher compared with lower protein on REE intake remained significant regardless of the difference in protein between the diets. However, the effect of higher compared with lower-protein intake was modified by BMI, sex, age, study design, blinding, energy balance, and study duration. The significant effect on REE was observed in studies with average BMIs <29.75 kg/m^2^, ≥35% male, average ages <35.71 y, parallel designs, single/double-blind designs, eucaloric diets and durations <28 d, but not in studies with BMIs ≥29.75 kg/m^2^, <35% male, ≥35 y, crossover designs, open-label designs, hypocaloric diets, and durations ≥28 d.

#### Postprandial substrate utilization

**FIGURE 6:**
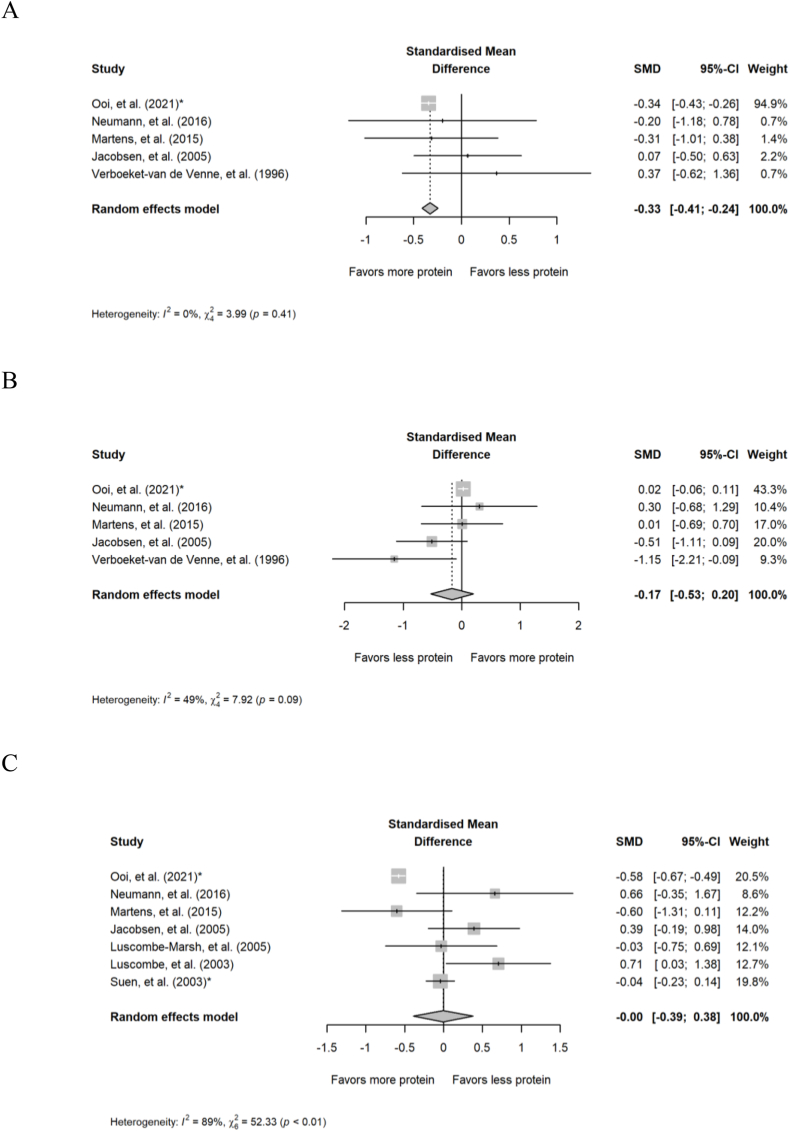
Forest plot of the effect of chronic diets with different amounts of protein on postprandial (A) carbohydrate oxidation, (B) fat oxidation, and (C) respiratory exchange ratio. CI, confidence interval; SMD, standardized mean difference. ∗Effect sizes for correlated comparisons within a study were pooled before running final analyses.

Five comparisons from 5 studies were included in the analyses of chronic intake of different amounts of protein on postprandial carbohydrate and fat oxidation. Diets containing higher protein reduced postprandial carbohydrate oxidation (SMD: –0.33; 95% CI: –0.41, –0.24; *P* < 0.001; Figure 6A) compared with diets containing lower protein, but there was no effect on postprandial fat oxidation (SMD: –0.17; 95% CI: –0.53, 0.20; *P =* 0.37; Figure 6B). There was no heterogeneity for the analysis of postprandial carbohydrate oxidation (*Q* = 3.99; *P =* 0.41; *I*^2^ = 0.00%) and moderate/high heterogeneity for the analysis of fat oxidation (Q = 7.92; *P =* 0.095; I^2^ = 49.5%). For postprandial RER, 7 comparisons from 7 studies demonstrated no effect of the amount of protein in the diet on postprandial RER (SMD: –0.003; 95% CI: –0.39, 0.38; *P =* 0.99; Figure 6C) with high heterogeneity (*Q* = 52.3; *P* < 0.001; *I*^2^ = 88.5). Findings from the subgroup analyses of substrate utilization in chronic studies are presented in Supplemental Table 3.

### Meta-analysis of acute meals or chronic diets containing different types of protein

The systematic review identified 15 studies that investigated the effect of different types or sources of protein on energy metabolism outcomes. These trials involved whey, casein, soy, gelatin, pork, salmon, veal, cod, dairy, mycoprotein, and/or chicken. To be included in the meta-analysis, there had to be ≥2 studies that shared a common active or comparator group. There was no effect of the whey content of the acute meals on DIT (SMD: 0.27; 95% CI: –0.11, 0.64; *P =* 0.17; *N* = 6), postprandial carbohydrate oxidation (SMD: 0.26; 95% CI: –0.01, 0.53; *P =* 0.06; *N* = 3), postprandial fat oxidation (SMD: –0.09; 95% CI: –0.43, 0.26; *P =* 0.62; *N* = 4), or RER (SMD: 0.79; 95% CI: –0.78, 2.37; *P =* 0.322; *N* = 3)**.** Additionally, there was no impact of acute meals containing red meat compared with fish on DIT (SMD: –0.23; 95% CI: –0.53, 0.07; *P =* 0.13; *N* = 2)**.** Finally, there was no impact of a chronic diet containing a mix of animal protein and vegetable protein compared with a chronic diet containing only vegetable protein on DIT (SMD: 0.15; 95% CI: –0.38, 0.66; *P =* 0.59; *N* = 3) or REE (SMD: –0.12; 95% CI: –0.38, 0.14; *P =* 0.38; *N* = 3). There were not enough studies with common active or comparator groups to conduct any other meta-analyses.

### Risk of bias and GRADE

The quality of the evidence, as assessed by GRADE criteria, for the analyses evaluating the effect of different amounts of protein is summarized in Supplemental Table 4. The quality of the evidence was rated as moderate for all energy metabolism outcomes in acute studies, except it was rated as low for the postprandial RER outcome. The rating of low was because of the risk of high heterogeneity and possible publication bias detected. Furthermore, the quality of the evidence was rated as low or very low for all energy metabolism outcomes in chronic studies. The rating of low or very low was because of the potential risk of bias, heterogeneity, and imprecision. Risk of bias assessment of randomized studies and funnel plots for the main outcomes in the analyses evaluating the effect of different amounts of protein are shown in Supplemental Table 5 and Supplemental Figures 2 and 3. Because the Cochrane risk of bias tool is only appropriate for randomized studies, the risk of bias for Schutz et al. [33] was assessed using the ROBINS-I Tool [18]. The risk of bias for this study was moderate because it was judged to be at moderate risk of bias for 1 domain (bias because of missing data) and low risk for bias for the other 6 domains. However, nonrandomized studies generally have higher risk of bias than randomized studies [23]. Small study effects were not evident for any outcomes, except possibly postprandial RER in acute studies, based on Egger’s regression (Supplemental Table 6). The funnel plot for postprandial RER in acute studies did not include studies in the lower right area of the funnel where smaller studies with nonsignificant studies would be located, suggesting potential publication bias (Supplemental Figure 2).

## Discussion

For the primary outcome of DIT, the meta-analysis demonstrated that acute meals containing higher compared with lower protein resulted in greater DIT, but chronic diets containing different amounts of protein did not impact DIT. Regarding TDEE, acute meals and chronic diets containing higher protein resulted in greater TDEE compared with lower-protein diets, but this effect was more pronounced for acute compared with chronic diets. Finally, chronic diets containing higher amounts of protein resulted in modestly higher REE compared with the diets containing lower amounts of protein.

The results suggest that TDEE increases in response to acute meals and chronic diets containing higher amounts of protein. TDEE comprises REE, DIT, and physical activity energy expenditure (PAEE) [78]. After acute consumption of meals higher in protein, it appears that the increase in DIT contributed to the increase in TDEE. After chronic consumption of a diet higher in protein, it appears that an increase in REE was the main factor contributing to higher TDEE, whereas the increase in DIT was not statistically significant. A potential explanation for this observation is that because of the body’s limited storage capacity for protein, acute protein-rich meals stimulate protein synthesis in skeletal muscle and other tissues, a process with high energy demands, resulting in higher DIT [79,80]. Other pathways involved in protein digestion, metabolism, and turnover may also contribute to the acute effect of higher protein intake on DIT [81]. Over time, higher-protein diets may result in greater fat-free mass, which is the primary determinant of REE [78,79]. Given that DIT was not increased with higher protein intake post meal after chronic consumption of a higher-protein diet, it is possible that there is an adaptation over time that limits the DIT response, although confirmation of this hypothesis would require additional studies. Regardless of the relative contributions of DIT, REE, and PAEE to the increase in TDEE, a small daily increase in energy expenditure may be clinically meaningful. Researchers estimate that an excess intake of 10–20 kcal/d relative to energy expenditure can explain the average yearly weight gain of 0.5–1.0 kg among United States adults, and a small annual weight gain can contribute to the development of obesity overtime [3,4]. The pooled SMD for TDEE in chronic studies was 0.31. For illustration, the study by Smeets et al. [61] had an SMD for the eucaloric conditions of 0.29, which is similar to the pooled SMD of 0.31 for chronic studies. The difference in TDEE in that trial between the higher and lower protein conditions was 72 kcal/d, or ∼3%. Therefore, the increase in energy expenditure produced by consumption of a protein-rich diet is large enough to contribute to reduced weight gain in adults if not fully offset by higher energy intake.

The subgroup analyses suggested that BMI may influence the impact of acute protein-rich meals on DIT. Acute meals containing higher protein increased DIT in the subgroup with BMI below the median of 23.8 kg/m^2^ but in the subgroup with BMI above the median. Five acute studies that were included in the meta-analysis directly compared the impact of higher with lower protein on DIT in participants with normal weight compared with overweight/obesity, and these studies reported conflicting findings [30,34,65,69,71]. Two of these studies reported no main effect of weight status or no intervention by BMI category interaction [30,34]. However, Labayen et al. [71] reported a significant main effect of weight status on DIT responses that suggested higher DIT in individuals with normal weight compared with obesity, and Riggs et al. [65] reported a tendency (*P =* 0.084) for a BMI by meal interaction that would support suppressed DIT in individuals with obesity. Finally, Bronstein et al. [69] reported a significant main effect of weight status on DIT when expressed in kJ, indicating that DIT was higher among women with overweight compared with normal weight, but this finding was no longer significant when DIT was expressed as a percentage of basal metabolic rate. Because these findings conflict with one other and somewhat contradict the results from our subgroup analysis, we conducted additional exploratory subgroup and sensitivity analyses to better understand if there is a true effect of weight status or if study design factors contributed to the meta-analysis results.

First, an exploratory subgroup analysis for BMI was conducted in crossover studies only, and the result was consistent with the BMI subgroup analysis that included both parallel and crossover studies. Next, sensitivity analyses were conducted in which similar studies were grouped together based on average BMI, protein difference, duration of DIT measurement, and meal size. Once again, meals higher in protein increased DIT in the subgroup analyses involving participants with BMIs in the normal weight category but not overweight/obesity categories. However, the borderline significant findings from these sensitivity analyses suggested that smaller measurement durations (<4 h) and larger meal sizes (≥550 kcal) may mask the effect of higher-protein meals on DIT in participants with overweight/obesity. Reed and Hill reported that individuals with normal weight have earlier and higher peaks for DIT when compared with individuals with obesity; thus, a shorter measurement duration would fail to capture a larger proportion of the DIT response in individuals with overweight or obesity [82]. Additionally, there may be a ceiling effect in which once the energy content of the meal reaches a certain size, DIT stops increasing, a hypothesis that is supported by the results of the sensitivity analyses [82]. Finally, when the measurement duration was graphed against the SMD in 17 of the studies that were included in the sensitivity analyses, the considerable variability in SMDs for the effects involving participants with overweight or obesity was illustrated. More specifically, 4 of the SMDs were < 0 and 5 were ≥0.

Regarding substrate utilization, acute meals containing higher compared with lower amounts of protein increased postprandial fat oxidation and decreased postprandial carbohydrate oxidation and RER. Notably, the heterogeneity between studies was substantial, so these results should be interpreted with caution. Previous research demonstrates that low rates of fat oxidation are predictive of weight gain [83,84]. Therefore, the favorable changes observed because of higher compared with lower-protein meals may contribute to weight management, although more long-term research is needed. In the control/referent groups, protein was replaced by carbohydrate, fat, or a combination of the 2. To better understand the impact of different types of replacements, the carbohydrate difference was calculated as the difference in percent of energy from carbohydrate in the lower-protein group minus the percent of energy from carbohydrate in the higher-protein group. In general, a larger carbohydrate difference indicated that mostly carbohydrate replaced protein in the lower-protein meal and that the protein difference between the higher and lower-protein meals was larger. Therefore, as expected, the magnitude of increase in postprandial fat and the magnitude of decrease in postprandial carbohydrate oxidation was much greater in the subgroups with larger carbohydrate differences (≥17.1% compared with <17.1%), which reflects the larger protein difference between the meals. In this meta-analysis, only 5 chronic studies reported postprandial macronutrient oxidation, which showed no change in postprandial fat oxidation and a reduction in postprandial carbohydrate oxidation.

There was no evidence that different types of protein impact energy metabolism. These results should be interpreted with caution because of the large degree of heterogeneity, small number of studies, and small samples sizes included in these studies. Regarding the meta-analysis of different amounts of protein, there was a large degree of heterogeneity between studies, and the subgroup analyses were often limited by the number and size of the studies. The subgroup analyses for different study designs showed greater heterogeneity between studies in the crossover design subgroup compared with the parallel design subgroup, especially for TDEE and REE in chronic studies. However, this observation is not straightforward to interpret. On average, there were tighter CIs for the crossover studies, indicating greater average precision of the individual point estimates, despite larger variation in the results across studies (Supplemental Figure 4). Because there is no obvious explanation for the greater heterogeneity of results within the category of crossover trials, additional research is needed to provide further insight. Additionally, the subgroup analyses did not account for the large variation in types of meals provided and methods for metabolism measurements. Finally, publication bias was difficult to assess for some outcomes because of the small number of studies, but there was an indication for potential publication bias for the analysis of postprandial RER in acute studies. The studies were mostly conducted in healthy subjects with or without obesity, so it is unclear if protein-rich meals/diets impact energy metabolism in populations with chronic diseases such as type 2 diabetes or cardiovascular disease.

Strengths of this systematic review and meta-analysis include the comprehensive literature search and the use of SMDs, which allowed for measurements of energy metabolism to be combined into a robust analysis despite different measurement methods and units. Additionally, the subgroup and sensitivity analyses generated greater insight into study design factors that may have contributed to the trial outcomes. Future studies should be undertaken to compare the impact of acute meals of moderate size (<550 kcal) containing a ≥20% difference in energy from protein on DIT (measured over ≥4 h) among participants with normal weights and overweight/obesity.

The results of the meta-analysis indicate that acute meals containing higher compared with lower protein resulted in greater DIT, but chronic diets containing different amounts of protein did not impact DIT. Furthermore, acute meals and chronic diets containing higher protein both resulted in greater TDEE compared with lower-protein diets, but this effect was more pronounced for acute meals. There was a modest increase in REE after chronic diets containing higher protein. Altogether, these results suggest that the increase in energy expenditure with higher-protein meals/diets may shift from DIT to REE over time, but more research is needed to confirm this hypothesis. The small increase in energy expenditure from consuming protein-rich meals and diets may mitigate the small daily energy imbalances, potentially impacting body weight trajectory.

## Author contributions

The authors’ responsibilities were as follows – LLG, BG-J, KK, KCM: designed research; LLG, CGA: conducted research; MLW: performed statistical analysis; LLG: wrote the manuscript; LLG, KCM: had primary responsibility for the final content; and all authors: read and approved the final manuscript.

## Funding

This research was funded by General Mills. The sponsor was not involved in the data collection, interpretation of data, or writing of the report. The sponsor collaborated with the authors during the conceptualization of the study, the analysis, and contributed to the decision to submit the manuscript for publication.

## Data availability

Data described in the manuscript and analytic code will be made available upon reasonable request.

## Conflict of interest

As employees of Midwest Biomedical Research, LLG, CGA, MLW, and KCM have received funding and/or consulting fees in the last 24 mo from 89bio, Inc., Acasti Pharma Inc., Beren Therapeutics, Bragg Live Products, Campbell’s, Cargill, Eli Lilly and Company, General Mills, Greenyn Biotechnology, Hass Avocado Board, Helaina Inc., Indiana University Foundation, Matinas BioPharma, Medifast, National Cattlemen’s Beef Association, National Dairy Council, Naturmega, New Amsterdam Pharma, NeuroEnergy VenturesPepsiCo, Pharmavite, and Seed. KK and BG-J are employees of General Mills and had a role in the conceptualization of the study, the analysis, and contributed to the decision to submit the manuscript for publication. The authors declare no additional conflicts of interest.
