# Supplementary material for: Effects of Varying Protein Amounts and Types on Diet-Induced Thermogenesis: A Systematic Review and Meta-Analysis
Source: Adv Nutr. 2024 Oct 31;15(12):100332. doi: 10.1016/j.advnut.2024.100332 (PMC11625215; doi:10.1016/j.advnut.2024.100332)
Supplement: multimedia component 1 [file mmc1.docx]

| **Supplemental Table 1. Sensitivity analyses investigating the acute effect of higher vs. lower protein meals on DIT in groupings of similar studies** | | | |
| --- | --- | --- | --- |
| **SMD (95% CI; P-value)** | **n** | **Q; P-value** | **I^2^ (%)** |
| **Chamber studies in participants with normal weights, protein differences of 15-20%, and 24-36 h measurements** | | | |
| 0.702 (0.147, 1.26); P = 0.013 | 4 | 10.1; P = 0.018 | 70.4 |
| **Studies in participants with normal weights and a protein difference of 10-20%** | | | |
| 0.673 (0.530, 0.816); P < 0.001 | 6 | 8.96; P = 0.111 | 44.2 |
| **Studies in participants with normal weights, protein differences of 10-20%, and meals ≥550 kcals** | | | |
| 0.623 (0.542, 0.704); P < 0.001 | 3 | 1.65; P = 0.439 | 0.00 |
| **Studies in participants with normal weights, protein differences of 10-20%, and meal <550 kcals** | | | |
| 0.828 (0.693, 0.963); P < 0.001 | 3 | 0.82; P = 0.664 | 0.00 |
| **Studies in participants with normal weights, protein differences of 10-20%, and a measurement duration ≥4 h** | | | |
| 0.708 (0.548, 0.867); P < 0.001 | 4 | 6.44; P = 0.0.92 | 53.4 |
| **Studies in participants with normal weights, protein differences of 10-20%, and a measurement duration <4 h** | | | |
| 0.493 (0.189, 0.797); P = 0.002 | 2 | 1.01; P = 0.316 | 0.54 |
| **Studies in participants with normal weights and protein differences of 26-45%** | | | |
| 0.589 (0.267, 0.911); P < 0.001 | 3 | 3.07; P = 0.215 | 35.0 |
| **Studies in participants with OW/OB and protein differences of 10-15%** | | | |
| -0.080 (-0.215, 0.055); P = 0.244 | 4 | 7.80; P = 0.050 | 61.5 |
| **Studies in participants with OW/OB and protein differences of 20-33%** | | | |
| 0.412 (-0.020, 0.843); P = 0.061 | 5 | 10.0; P = 0.040 | 60.1 |
| **Studies in participants with OW/OB, protein differences of 20-33%, and meals ≥550 kcals** | | | |
| 0.316 (-0.920, 1.55); P = 0.617 | 2 | 6.13; P = 0.013 | 83.7 |
| **Studies in participants with OW/OB, protein differences of 20-33%, and meals <550 kcals** | | | |
| 0.436 (-0.058, 0.930); P = 0.084 | 3 | 3.62; P = 0.164 | 44.8 |
| **Studies in participants with OW/OB, protein differences of 20-33%, and a measurement duration ≥4 h** | | | |
| 0.592 (-0.020, 1.20); P = 0.058 | 3 | 7.03; P = 0.030 | 71.5 |
| **Studies in participants with OW/OB, protein differences of 20-33%, and a measurement duration < 4 h** | | | |
| 0.134 (-0.871, 1.14); P = 0.794 | 2 | 2.66; P = 0.103 | 62.4 |
| The protein difference represents the difference in percent of energy from protein in the higher and lower protein groups.  Studies with participants with normal weight had an average body mass index (BMI) <25 kg/m^2^. Studies with participants with OW/OB had an average BMI > 25 kg/m^2^.  Individual effect sizes within each grouping were pooled using random effects models that utilized inverse-variance weighting and the DerSimonian-Laird estimator. Correlated, within-study comparisons that fell in the same grouping were pooled prior to running each analysis. Heterogeneity within groupings was assessed using Cochran’s Q and the I^2^ statistic (low heterogeneity: 0-40%; moderate/higher heterogeneity: >40%).  Abbreviation: OW/OB = overweight or obesity. | | | |

| **Supplemental Table 2.** Subgroup analyses of the effect of acute intake of higher vs. lower protein meals on postprandial substrate utilization | | | | |
| --- | --- | --- | --- | --- |
| **Outcome and Subgroups** | **n** | **SMD (95% CI); p-value** | **Q; p-value** | **I^2^ (%)** |
| **Postprandial Carbohydrate Oxidation** | | | | |
| Protein Difference (median split)^1^ | | | | |
| <19.8% | 10 | -0.498 (-0.835, -0.161); P = 0.004 | 159; P < 0.001 | 94.3 |
| ≥19.8% | 11 | -0.474 (-0.966, 0.017); P = 0.059 | 143; P < 0.001 | 93.0 |
| % Male (median split) | | | | |
| <51.3% | 10 | -0.518 (-0.873, -0.162); P = 0.004 | 161; P < 0.001 | 94.4 |
| ≥51.3% | 8 | -0.580 (-1.04, -0.118); P = 0.014 | 542; P < 0.001 | 98.7 |
| BMI (kg/m^2^) (median split) | | | | |
| <26.4 | 9 | -0.681 (-1.10, -0.262); P = 0.001 | 161; P < 0.001 | 95.0 |
| ≥26.4 | 9 | -0.440 (-0.914, 0.035); P = 0.069 | 657; P < 0.001 | 98.8 |
| Age (y) (median split) | | | | |
| <26.1 | 8 | -0.812 (-1.48, -0.146); P = 0.017 | 347; P < 0.001 | 98.0 |
| ≥26.1 | 8 | -0.401 (-0.752, -0.049); P = 0.026 | 361; P < 0.001 | 98.1 |
| Study Design | | | | |
| Parallel | 6 | -0.485 (-0.941, -0.029); P = 0.037 | 11.5; P = 0.042 | 56.7 |
| Crossover | 12 | -0.589 (-0.910, -0.267); P<0.001 | 593; P < 0.001 | 98.1 |
| Blinding | | | | |
| Single/Double | 10 | -0.568 (-0.945, -0.191); P = 0.003 | 693; P < 0.001 | 98.7 |
| Open-label | 8 | -0.528 (-0.954, -0.102); P = 0.015 | 55.5; P < 0.001 | 87.4 |
| Carbohydrate Difference (median split)^2^ | | | | |
| <17.1% | 10 | -0.316 (-0.639, 0.007); P = 0.055 | 153; P < 0.001 | 94.1 |
| ≥17.1% | 11 | -0.658 (-1.15, -0.162); P = 0.009 | 132; P < 0.001 | 92.4 |
| Risk of Bias |  |  |  |  |
| Low | 15 | -0.578 (-0.891, -0.264); P < 0.001 | 595; P < 0.001 | 97.6 |
| High/Some concerns | 3 | -0.443 (-1.07, 0.180); P = 0.163 | 42.9; P < 0.001 | 95.3 |
| **Postprandial Fat Oxidation** | | | | |
| Protein Difference (median split)^1^ | | | | |
| <19.8% | 10 | 0.304 (0.128; 0.480); P < 0.001 | 36.9; P < 0.001 | 75.6 |
| ≥19.8% | 11 | 0.486 (0.169, 0.804); P = 0.003 | 66.1; P < 0.001 | 84.9 |
| % Male (median split) | | | | |
| <51.3% | 10 | 0.339 (-0.079, 0.757); P = 0.112 | 245; P < 0.001 | 96.3 |
| ≥51.3% | 8 | 0.059 (-0.410, 0.528); P = 0.805 | 551; P < 0.001 | 98.7 |
| BMI (kg/m^2^) (median split) | | | | |
| <26.4 | 9 | 0.491 (0.027, 0.954); P = 0.038 | 210; P < 0.001 | 96.2 |
| ≥26.4 | 9 | -0.023 (-0.502, 0.456); P = 0.925 | 675; P < 0.001 | 98.8 |
| Age (y) (median split) | | | | |
| <26.1 | 8 | 0.615 (0.002, 1.23); P = 0.049 | 308; P < 0.001 | 97.7 |
| ≥26.1 | 8 | -0.260 (-0.626, 0.107); P = 0.165 | 392; P < 0.001 | 98.2 |
| Study Design | | | | |
| Parallel | 6 | 0.554 (-0.489, 1.60); P = 0.298 | 61.1; P < 0.001 | 91.8 |
| Crossover | 12 | 0.116 (-0.208, 0.439); P = 0.483 | 612; P < 0.001 | 98.2 |
| Blinding | | | | |
| Single/Double | 10 | 0.119 (-0.267, 0.504); P = 0.547 | 726; P < 0.001 | 98.8 |
| Open-label | 8 | 0.376 (-0.262, 1.02); P = 0.248 | 147; P < 0.001 | 95.2 |
| Carbohydrate Difference (median split)^2^ | | | | |
| <17.1% | 10 | 0.179 (0.061, 0.297); P = 0.003 | 17.5; P = 0.042 | 48.5 |
| ≥17.1% | 11 | 0.623 (0.287, 0.959); P<0.001 | 68.6; P < 0.001 | 85.4 |
| Risk of Bias |  |  |  |  |
| Low | 15 | 0.566 (0.319, 0.813); P < 0.001 | 394; P < 0.001 | 96.4 |
| High/Some concerns | 3 | 0.169 (-0.001, 0.339); P = 0.051 | 3.56; P = 0.169 | 43.8 |
| **Postprandial Respiratory Exchange Ratio** | | | | |
| Protein Difference (median split)^1^ | | | | |
| <19.5% | 11 | -0.419 (-0.638, -0.199); P < 0.001 | 204; P < 0.001 | 95.1 |
| ≥19.5% | 17 | -0.472 (-0.869, -0.075); P = 0.020 | 230; P < 0.001 | 93.0 |
| % Male (median split) | | | | |
| <45% | 15 | -0.484 (-0.796, -0.172); P = 0.002 | 87.4; P < 0.001 | 84.0 |
| ≥45% | 11 | -0.459 (-0.709, -0.210); P < 0.001 | 701; P < 0.001 | 98.6 |
| BMI (kg/m^2^) (median split) | | | | |
| <23.8 | 13 | -0.337 (-0.566, -0.107); P = 0.004 | 225; P < 0.001 | 94.7 |
| ≥23.8 | 13 | -0.556 (-0.780, -0.331); P < 0.001 | 274; P < 0.001 | 95.6 |
| Age (y) (median split) | | | | |
| <26.1 | 13 | -0.607 (-0.838, -0.376); P < 0.001 | 236; P < 0.001 | 94.9 |
| ≥26.1 | 13 | -0.315 (-0.532, -0.099); P = 0.004 | 245; P < 0.001 | 95.1 |
| Study Design | | | | |
| Parallel | 5 | -0.713 (-1.08, -0.342); P < 0.001 | 7.83; P = 0.098 | 48.9 |
| Crossover | 21 | -0.396 (-0.563, -0.229); P < 0.001 | 509; P < 0.001 | 96.1 |
| Blinding | | | | |
| Single/Double | 17 | -0.423 (-0.625, -0.221); P < 0.001 | 724; P < 0.001 | 97.8 |
| Open-label | 9 | -0.511 (-1.02, -0.003) P = 0.049 | 51.6; P < 0.001 | 84.5 |
| Carbohydrate Difference (median split)^2^ | | | | |
| <15% | 10 | -0.093 (-0.409, 0.223); P = 0.563 | 506; P < 0.001 | 98.2 |
| ≥15% | 18 | -0.715 (-0.996, -0.434); P < 0.001 | 115; P < 0.001 | 85.2 |
| Risk of Bias |  |  |  |  |
| Low | 19 | -0.462 (-0.638, -0.285); P < 0.001 | 334; P < 0.001 | 94.6 |
| High/Some concerns | 7 | -0.365 (-0.652, -0.078); P = 0.013 | 92.4; P < 0.001 | 93.5 |
| ^1^The protein difference represents the difference in percent of energy from protein in the higher and lower protein groups. ^2^The carbohydrate difference was calculated as the difference in percent of energy from carbohydrate in the lower protein group minus the percent of energy from carbohydrate in the higher protein group.  Individual effect sizes within each subgroup were pooled using random effects models that utilized inverse-variance weighting and the DerSimonian-Laird estimator. Correlated, within-study comparisons that fell in the same subgroup were pooled prior to running each analysis. Heterogeneity within groupings was assessed using Cochran’s Q and the I^2^ statistic (low heterogeneity: 0-40%; moderate/higher heterogeneity: >40%).  BMI = body mass index; NW/UW=normal weight/underweight; OW/OB=overweight/obese | | | | |

| **Supplemental Table 3.** Subgroup analysis of the effect of chronic intake of higher vs. lower protein meals on postprandial substrate utilization | | | | |
| --- | --- | --- | --- | --- |
| **Outcome and Subgroup** | **n** | **SMD (95% CI); p-value** | **Q; p-value** | **I^2^ (%)** |
| **Postprandial Carbohydrate Oxidation** | | | | |
| Protein Difference (median split)^1^ | | | | |
| <13% | - | -- | -- | -- |
| ≥13% | 3 | -0.342 (-0.428, -0.257); P < 0.001 | 0.09; P = 0.956 | 0.00 |
| % Male (median split) | | | | |
| <47% | 3 | -0.102 (-0.502, 0.298); P = 0.616 | 0.73; P = 0.694 | 0.00 |
| ≥47% | - | -- | -- | -- |
| BMI (kg/m^2^) (median split) | | | | |
| <26.4 | 3 | -0.115 (-0.608, 0.379); P = 0.649 | 1.26; P = 0.534 | 0.00 |
| ≥26.4 | - | -- | -- | -- |
| Age (y) (median split) | | | | |
| <25.35 | 3 | -0.102 (-0.502, 0.298); P = 0.616 | 0.73; P = 0.694 | 0.00 |
| ≥25.35 | - | -- | -- | -- |
| Study Design | | | | |
| Parallel | 4 | -0.337 (-0.422, -0.252); P < 0.001 | 2.06; P = 0.560 | 0.00 |
| Crossover | - | -- | -- | -- |
| Blinding | | | | |
| Single/Double | 3 | -0.342 (-0.428, -0.257); P < 0.001 | 0.09; P = 0.956 | 0.00 |
| Open-label | - | -- | -- | -- |
| Carbohydrate difference (median split)^2^ | | | | |
| <13% | - | -- | -- | -- |
| ≥13% | 3 | -0.342 (-0.428, -0.257); P < 0.001 | 0.09; P = 0.956 | 0.00 |
| Energy Balance |  |  |  |  |
| Hypocaloric | -- | -- | -- | -- |
| Eucaloric | 3 | -0.009 (-0.410, 0.391); P = 0.964 | 1.36; P = 0.507 | 0.00 |
| Hypercaloric | -- | -- | -- | -- |
| Duration of Intervention (medial split) | | | | |
| <98 days | 3 | -0.102 (-0.502, 0.298); P = 0.616 | 0.73; P = 0.694 | 0.00 |
| ≥98 days | -- | -- | -- | -- |
| Risk of Bias |  |  |  |  |
| Low | -- | -- | -- | -- |
| High/Some concerns | 3 | -0.338 (-0.424, -0.253); P<0.001 | 1.98; P = 0.371 | 0.00 |
| **Postprandial Fat Oxidation** | | | | |
| Protein Difference (median split)^1^ | | | | |
| <13% | -- | -- | -- | -- |
| ≥13% | 3 | 0.026 (-0.058, 0.109); P = 0.545 | 0.31; P = 0.858 | 0.00 |
| % Male (median split) | | | | |
| <47% | 3 | -0.168 (-0.622, 0.285); P = 0.466 | 2.33; P = 0.311 | 14.3 |
| ≥47% | -- | -- | -- | -- |
| BMI (kg/m^2^) (median split) | | | | |
| <26.4 | 3 | -0.234 (-1.01, 0.543); P = 0.555 | 4.44; P = 0.109 | 54.9 |
| ≥26.4 | -- | -- | -- | -- |
| Age (y) (median split) | | | | |
| <25.35 | 3 | -0.168 (-0.622, 0.285); P = 0.466 | 2.33; P = 0.311 | 14.3 |
| ≥25.35 | -- | -- | -- | -- |
| Study Design | | | | |
| Parallel | 4 | -0.284 (-0.660, 0.093); P = 0.140 | 4.86; P = 0.183 | 38.2 |
| Crossover | -- | -- | -- | -- |
| Blinding | | | | |
| Single/Double | 3 | -0.247 (-0.530, 0.035); P = 0.086 | 2.57; P = 0.276 | 22.2 |
| Open-label | -- | -- | -- | -- |
| Carbohydrate difference (median split)^2^ | | | | |
| <13% | - | -- | -- | -- |
| ≥13% | 3 | 0.026 (-0.058, 0.109); P = 0.545 | 0.31; P = 0.858 | 0.00 |
| Energy Balance | | | | |
| Hypocaloric | -- | -- | -- | -- |
| Eucaloric | 3 | -0.457 (-1.02, 0.108); P = 0.113 | 3.37; P = 0.186 | 40.6 |
| Hypercaloric | -- | -- | -- | -- |
| Duration of Intervention (days) (median split) | | | | |
| <98 | 3 | -0.168 (-0.622, 0.285); P = 0.466 | 2.33; P = 0.311 | 14.3 |
| ≥98 | -- | -- | -- | -- |
| Risk of Bias |  |  |  |  |
| Low | -- | -- | -- | -- |
| High/Some concerns | 3 | -0.176 (-0.689, 0.337); P = 0.500 | 1.89; P = 0.094 | 57.6 |
| **Postprandial Respiratory Exchange Ratio** | | | | |
| Protein Difference (median split)^1^ | | | | |
| <21% | 3 | -0.122 (-0.793, 0.549); P = 722 | 12.3; P = 0.002 | 83.8 |
| ≥21% | 4 | 0.117 (-0.388, 0.621); P = 0.651 | 8.83; P = 0.032 | 66.0 |
| % Male (median split) | | | | |
| <28% | 3 | -0.162 (-0.228, 0.553); P = 0.415 | 3.53; P = 0.171 | 43.4 |
| ≥28% | 4 | -0.167 (-0.760, 0.427); P = 0.582 | 15.8; P = 0.001 | 81.0 |
| BMI (kg/m^2^) (median split) | | | | |
| <29.75 | 3 | 0.116 (-0.628, 0.860); P = 0.760 | 5.91; P = 0.052 | 66.1 |
| ≥29.75 | 4 | -0.068 (-0.532, 0.397); P = 0.776 | 39.6; P < 0.001 | 92.4 |
| Age (y) (median split) | | | | |
| <32.9 | 4 | 0.115 (-0.731, 0.501); P = 0.714 | 15.9; P = 0.001 | 81.1 |
| ≥32.9 | 3 | 0.142 (-0.292, 0.576); P = 0.520 | 4.41; P = 0.110 | 54.6 |
| Study Design | | | | |
| Parallel | 5 | -0.039 (-0.621, 0.544); P = 0.897 | 21.3; P < 0.001 | 81.2 |
| Crossover | -- | -- | -- | -- |
| Blinding | | | | |
| Single/Double | 4 | -0.248 (-0.688, 0.192); P = 0.269 | 31.6; P < 0.001 | 90.5 |
| Open-label | 3 | 0.373 (-0.019, 0.764); P = 0.062 | 2.15; P = 0.341 | 7.11 |
| Carbohydrate difference (median split)^2^ | | | | |
| <13% | 3 | 0.102 (-0.275, 0.479); P = 0.596 | 1.67; P = 0.433 | 0.00 |
| ≥13% | 5 | -0.014 (-0.604, 0.575); P = 0.963 | 22.7; P < 0.001 | 82.3 |
| Energy Balance | | | | |
| Hypocaloric | 3 | -0.072 (-0.597, 0.453); P = 0.787 | 38.3; P < 0.001 | 94.8 |
| Eucaloric | 3 | -0.054 (-0.633, 0.525); P = 0.855 | 4.49; P = 0.106 | 55.4 |
| Hypercaloric | -- | -- | -- | -- |
| Duration of Intervention (days) (median split) | | | | |
| <84 | 3 | 0.162 (-0.228, 0.553); P = 0.415 | 3.53; P = 0.171 | 43.4 |
| ≥84 | 4 | -0.167 (-0.760, 0.427); P = 0.582 | 15.8; P = 0.001 | 81.0 |
| Risk of bias |  |  |  |  |
| Low | 4 | 0.409 (0.056, 0.762); P = 0.023 | 2.43; P = 0.489 | 0.00 |
| High/Some concerns | 3 | -0.375 (-0.823, 0.072); P = 0.100 | 26.7; P < 0.001 | 92.5 |
| ^1^The protein difference represents the difference in percent of energy from protein in the higher and lower protein groups. ^2^The carbohydrate difference was calculated as the difference in percent of energy from carbohydrate in the lower protein group minus the percent of energy from carbohydrate in the higher protein group.  Rows without data did not have enough comparisons (< 3) to perform a subgroup analysis.  Individual effect sizes within each subgroup were pooled using random effects models that utilized inverse-variance weighting and the DerSimonian-Laird estimator. Correlated, within-study comparisons that fell in the same subgroup were pooled prior to running each analysis. Heterogeneity within groupings was assessed using Cochran’s Q and the I^2^ statistic (low heterogeneity: 0-40%; moderate/higher heterogeneity: >40%). BMI = body mass index; NW/UW=normal weight/underweight; OW/OB=overweight/obese | | | | |

| **Supplemental Table 4.** Quality of evidence included in the systematic review and meta-analysis investigating the impact of consuming meals/diets containing different amounts of protein on energy metabolism, based on GRADE approach | | | | | | | |
| --- | --- | --- | --- | --- | --- | --- | --- |
| **Outcome** | **Number of Effects/ Studies/ Participants** | **Within-Study Risk of Bias*** | **Indirectness** | **Inconsistency** | **Imprecision** | **Publication Bias** | **Quality of Evidence** |
| ***Acute Studies*** |  |  |  |  |  |  |  |
| DIT | 32/27/636 | Minor limitations (no downgrade): Of 28 studies, 75% were rated as “low” risk of bias. Six studies (21%) were rated as “some concerns” for risk of bias, and one study (4%) was rated as “high” risk of bias due to an inadvertent dietary treatment mistake for 8 of 46 participants. | Direct | Moderate/high heterogeneity; I^2^=97.7% (-1) | Precise^2^ | Publication bias undetected | ●●●○  Moderate |
| TDEE | 9/9/190 | Minor limitations (no downgrade): One of 9 studies (11%) were rated as “some concerns” for risk of bias due to not enough information about reasons for missing data. The rest were rated as “low” risk of bias. | Direct | Moderate/high heterogeneity; I^2^=64.6% (-1) | Precise^2^ | Publication bias undetected^1^ | ●●●○  Moderate |
| PP carbohydrate oxidation | 18/16/456 | Minor limitations (no downgrade): Of 16 studies, two studies (13%) were rated as “some concerns” for risk of bias, and one study (6%) was rated as “high” for risk of bias. The rest were rated as “low” risk of bias. | Direct | Moderate/high heterogeneity; I^2^=97.9% (-1) | Precise^2^ | Publication bias undetected | ●●●○  Moderate |
| PP fat oxidation | 18/16/456 | Minor limitations (no downgrade): Of 16 studies, two studies (13%) were rated as “some concerns” for risk of bias, and one study (6%) was rated as “high” for risk of bias. The rest were rated as “low” risk of bias. | Direct | Moderate/high heterogeneity; I^2^=95.8% (-1) | Precise^2^ | Publication bias undetected | ●●●○  Moderate |
| PP RER | 25/23/580 | Minor limitations (no downgrade): Of 24 studies, six studies (25%) were rated as “some concerns” for risk of bias, and one study (4%) was rated as “high” for risk of bias. The rest were rated as “low” risk of bias. | Direct | Moderate/high heterogeneity; I^2^=97.1% (-1) | Precise^2^ | Possible publication bias detected (-1) | ●●○○  Low |
| ***Chronic Studies*** |  |  |  |  |  |  |  |
| DIT | 14/13/340 | Potential limitations (-1): Of 13 studies, 12 were RCTs. Five of the 12 RCTs (42%) were rated as “some concerns” for risk of bias. The remaining RCTs were rated as “low” risk of bias. The NRT was rated as “moderate” risk of bias (due to missing data) using the ROBINS-I tool. We downgraded by one level due to the large proportion of studies with “some concerns” or “fair” ratings. | Direct | Moderate/high heterogeneity; I^2^=94.2% (-1) | Imprecise^3^ (-1) | Publication bias undetected | ●○○○  Very Low |
| TDEE | 10/10/280 | Potential limitations (-1): Four out of 10 studies (40%) were rated as “some concerns” for risk of bias, and the rest were “low” risk of bias. We downgraded by one level due to the large proportion of studies with “some concerns”. | Direct | Moderate/high heterogeneity; I^2^=80.5% (-1) | Imprecise^3^ (-1) | Publication bias undetected | ●○○○  Very Low |
| REE | 13/12/400 | Potential limitations (-1): Of 12 studies, 11 were RCTs. Four of the 11 RCTs (36%) were rated as “some concerns” for risk of bias. The remaining RCTs were rated as “low” risk of bias. The NRT was rated as “moderate” risk of bias (due to missing data) using the ROBINS-I tool. We downgraded by one level due to the large proportion of studies with “some concerns” or “fair” ratings. | Direct | Moderate/high heterogeneity; I^2^=93.2% (-1) | Imprecise^3^ (-1) | Publication bias undetected | ●○○○  Very Low |
| PP carbohydrate oxidation | 5/5/183 | Potential limitations (-1): Three out of 5 studies (60%) were rated as “some concerns” for risk of bias, and the rest were “low” risk of bias. We downgraded by one level due to the large proportion of studies with “some concerns”. | Direct | No heterogeneity; I^2^=0.00% | Imprecise^3^ (-1) | Publication bias undetected^1^ | ●●○○  Low |
| PP fat oxidation | 5/5/183 | Potential limitations (-1): Three out of 5 studies (60%) were rated as “some concerns” for risk of bias, and the rest were “low” risk of bias. We downgraded by one level due to the large proportion of studies with “some concerns”. | Direct | Moderate heterogeneity; I^2^=49.5% (-1) | Imprecise^3^ (-1) | Publication bias undetected^1^ | ●○○○  Very Low |
| PP RER | 7/7/240 | Potential limitations (-1): Three out of 7 studies (43%) were rated as “some concerns” for risk of bias, and the rest were “low” risk of bias. We downgraded by one level due to the large proportion of studies with “some concerns”. | Direct | Moderate/high heterogeneity; I^2^=88.5% (-1) | Imprecise^3^ (-1) | Publication bias undetected^1^ | ●○○○  Very Low |
| *The Cochrane Risk of Bias-2 Tool was used to assess risk of bias for RCTs and the Risk of Bias in Non-Randomized Studies of Interventions (ROBINS-I) Tool was used to assess risk of bias for the NRT.  ^1^ Publication bias for <10 effects is difficult to assess but was not downgraded because a thorough systematic search was conducted. ^2^ OIS was met, and 95% confidence interval excludes no effect. ^3^OIS was not met.  **Abbreviations:** CI = confidence interval; DIT=diet induced thermogenesis; GRADE=Grading of Recommendations Assessment, Development and Evaluation; NRT=nonrandomized trial; OIS = optimal information size; PP=postprandial; RCT=randomized controlled trial; RER=respiratory exchange ratio; TDEE=total daily energy expenditure. | | | | | | | |

| **Supplemental Table 5.** Risk of bias assessment of randomized trials | | | | | | | | |
| --- | --- | --- | --- | --- | --- | --- | --- | --- |
| **First author, Year** | **Outcome** | **Randomization Process** | **Bias Arising from Period/Carryover Effects (Crossover Studies Only)** | **Deviation from Intended Interventions** | **Missing Outcome Data** | **Measurement of the Outcome** | **Selection of the Reported Result** | **Overall Bias** |
| Abdouni, et al. (2018) | DIT | Low | N/A | Low | Low | Low | Low | Low |
| Acheson, et al. (2011) | DIT | Low | Low | Low | Low | Low | Low | Low |
| Barnard, et al. (2005) | DIT | Low | N/A | Low | Low | Low | Low | Low |
| Batterham, et al. (2008) | DIT | Low | Low | Low | Low | Low | Low | Low |
| Bellissimo, et al. (2020) | DIT | Low | Low | Low | Low | Low | Low | Low |
| Bendtsen, et al. (2014) | DIT | Low | Low | Low | Low | Low | Low | Low |
| Bottin, et al. (2016) | DIT | Low | Low | Low | Low | Low | Low | Low |
| Bray, et al. (2012) | TDEE | Low | N/A | Some Concerns | Some Concerns | Low | Low | Some Concerns |
| Bray, et al. (2015) | TDEE | Low | N/A | Low | Low | Low | Low | Low |
| Brehm, et al. (2005) | DIT | Low | Some Concerns | Low | Low | Low | Low | Some Concerns |
| Bronstein, et al. (1995) | DIT | Low | Low | Low | Low | Low | Low | Low |
| Crovetti, et al. (1997) | DIT | Low | Low | Low | Low | Low | Low | Low |
| Das, et al. (2008) | TDEE | Low | N/A | Low | Low | Low | Low | Low |
| Gentile, et al. (2015) | DIT | Low | Low | Low | Low | Low | Low | Low |
| Hochstenback-Waelen, et al. (2009) | DIT | Low | Low | Low | Low | Low | Low | Low |
| Hursel, et al. (2010) | DIT | Low | Low | Low | Low | Low | Low | Low |
| Jacobsen, et al. (2005) | TDEE | Low | Low | Low | Low | Low | Low | Low |
| Johnston, et al. (2002) | DIT | Low | Low | Low | Low | Low | Some Concerns | Some Concerns |
| Kassis, et al. (2019) | DIT | Low | Low | Low | Low | Low | Low | Low |
| Labayen, et al. (2004) | DIT | Low | N/A | Low | Low | Low | Low | Low |
| Leidy, et al. (2007) | DIT | Low | Low | High | Low | Low | Low | High |
| Lejeune, et al. (2006) | DIT | Low | Low | Low | Low | Low | Low | Low |
| Li, et al. (2016) | DIT | Low | N/A | Low | Low | Low | Low | Low |
| Lorenzen, et al. (2012) | DIT | Low | Low | Low | Low | Low | Low | Low |
| Luscombe, et al. (2003) | DIT | Low | N/A | Low | Low | Low | Low | Low |
| Luscombe-Marsh, et al. (2005) | DIT | Low | N/A | Low | Low | Low | Low | Low |
| Martens, et al. (2015) | DIT | Low | N/A | Some Concerns | Low | Low | Low | Some Concerns |
| Mikkelsen, et al. (2000) | DIT | Low | Low | Low | Low | Low | Low | Low |
| Neumann, et al. (2019) | DIT | Low | N/A | Low | Low | Low | Low | Low |
| Nguo, et al. (2019) | DIT | Low | Low | Low | Low | Low | Low | Low |
| Nielsen, et al. (2018) | DIT | Low | Low | Low | Low | Low | Low | Low |
| Nielsen, et al. (2019) | DIT | Low | Low | Low | Low | Low | Low | Low |
| Oliveira, et al. (2021) | TDEE | Low | Low | Low | Low | Low | Low | Low |
| Ooi, et al. (2021) | DIT | Low | N/A | Some Concerns | Low | Low | Low | Some Concerns |
| Raben, et al. (2014) | DIT | Low | Low | Low | Low | Low | Low | Low |
| Riggs, et al. (2007) | DIT | Low | Low | Low | Low | Low | Low | Low |
| Sambashivaiah, et al. (2023) | DIT | Low | Low | Low | Low | Low | Low | Low |
| Scott, et al. (2005) | DIT | Low | Some Concerns | Low | Low | Low | Low | Some Concerns |
| Smeets, et al. (2008) | DIT | Low | Some Concerns | Low | Low | Low | Low | Some Concerns |
| Smeets, et al. (2013) | DIT | Low | Low | Low | Some Concerns | Low | Low | Some Concerns |
| Stiegler, et al. (2008) | DIT | Low | N/A | Low | Low | Low | Low | Low |
| Suen, et al. (2003) | DIT | Low | Low | Low | Some Concerns | Low | Low | Some Concerns |
| Surowska, et al. (2019) | DIT | Low | Low | Low | Low | Low | Low | Low |
| Tan, et al. (2010) | DIT | Low | Low | Low | Low | Low | Low | Low |
| Veldhorst, et al. (2010) | TDEE | Low | Low | Low | Low | Low | Low | Low |
| Verboeket-van de Venn, et al. (1996) | DIT | Low | N/A | Some Concern | Low | Low | Low | Some Concern |
| Walsh, et al. (2013) | DIT | Low | Low | Some Concerns | Low | Low | Low | Some Concerns |
| Westerterp, et al. (1999) | DIT | Low | Low | Low | Low | Low | Low | Low |
| Westerterp-Plantenga, et al. (2009) | DIT | Low | Low | Low | Low | Low | Low | Low |
| Whitehead, et al. (1996) | TDEE | Low | Low | Low | Low | Low | Low | Low |
| Xiong, et al. (2022) | DIT | Low | Low | Low | Low | Low | Low | Low |
| DIT = diet induced thermogenesis; TDEE = total daily energy expenditure. | | | | | | | | |

| **Supplemental Table 6.** Analysis of small study effects for studies evaluating the effect of meals and diets containing different amounts of protein on energy metabolism | | |
| --- | --- | --- |
|  | **Acute meals/diets** | **Chronic Diets** |
| **Outcome** | **P-Value^^^** | **P-Value^** |
| Diet Induced Thermogenesis | 0.740 | 0.551 |
| Total Daily Energy Expenditure | 0.694* | 0.591 |
| Resting Energy Expenditure | N/A | 0.877 |
| Postprandial Carbohydrate Oxidation | 0.274 | 0.121* |
| Postprandial Fat Oxidation | 0.098 | 0.296* |
| Postprandial Respiratory Exchange Ratio | 0.030 | 0.067* |
| ^Egger’s Regression  *Interpret with caution due to <10 effects. | | |

**A**

**B**

**C**

**Supplemental Figure 1.** Standardized mean difference by measurement duration in (A) all studies, (B), studies involving participants with normal weight [body mass index (BMI) <25 kg/m^2^], and (C) studies involving participants with overweight or obesity (BMI ≥25 kg/m^2^).

**Supplemental Figure 2.** Contour-enhanced funnel plot for analyses of the effect of acute meals and diets containing different amounts of protein on (A) diet induced thermogenesis (B) total daily energy expenditure, (C) postprandial carbohydrate oxidation, (D) postprandial fat oxidation, and (E) postprandial respiratory exchange ratio. Shaded regions represent varying levels of statistical significance.

A B C


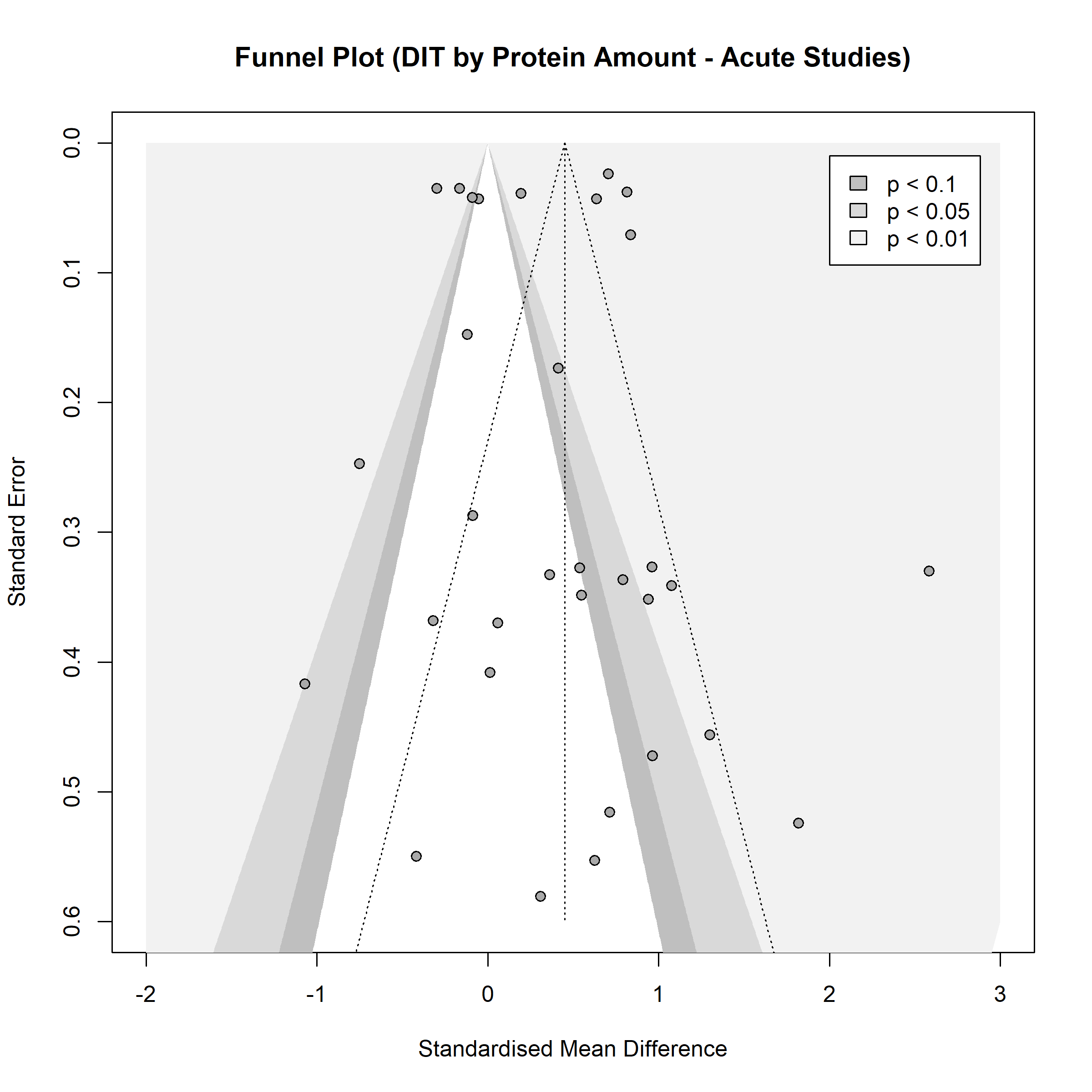

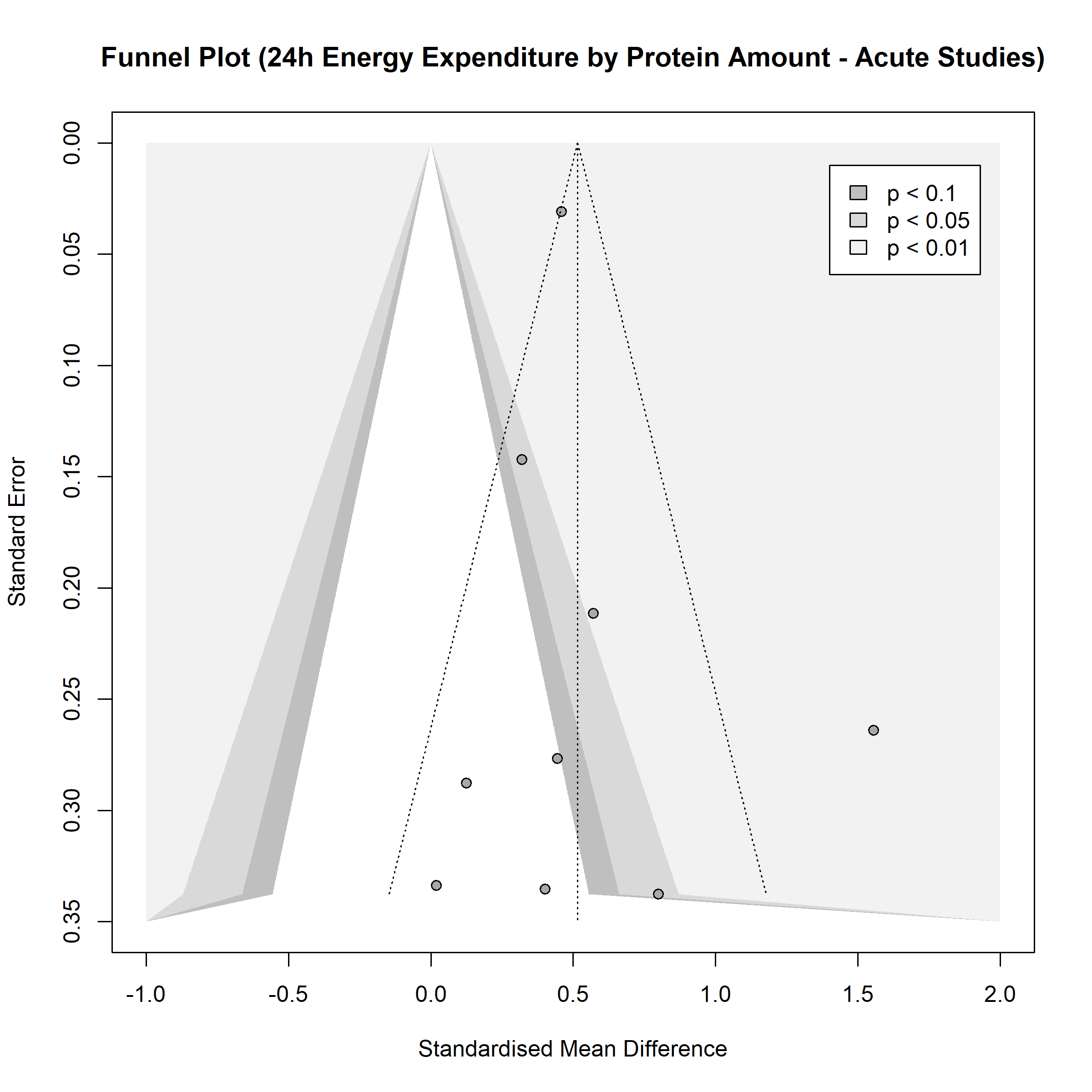

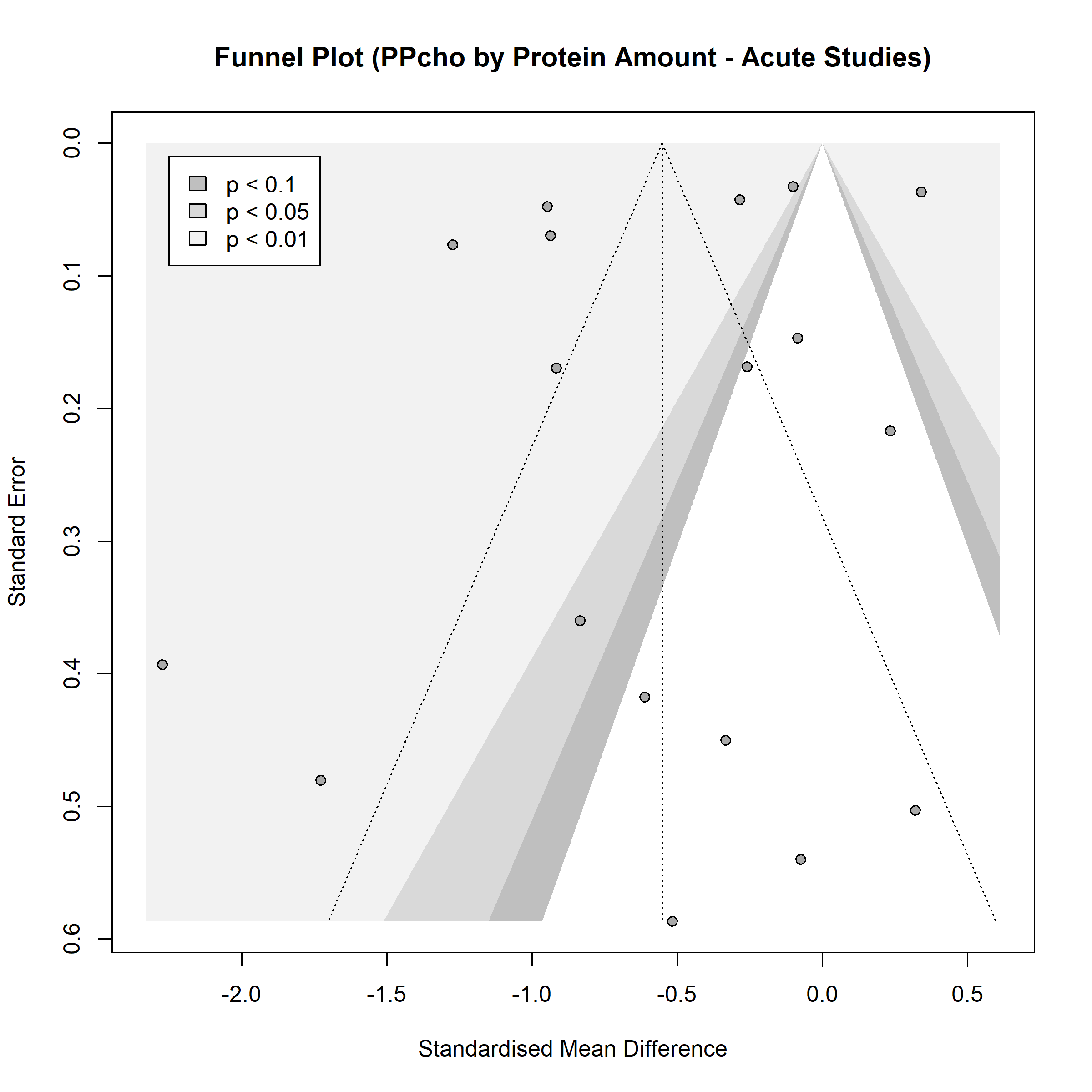


D E


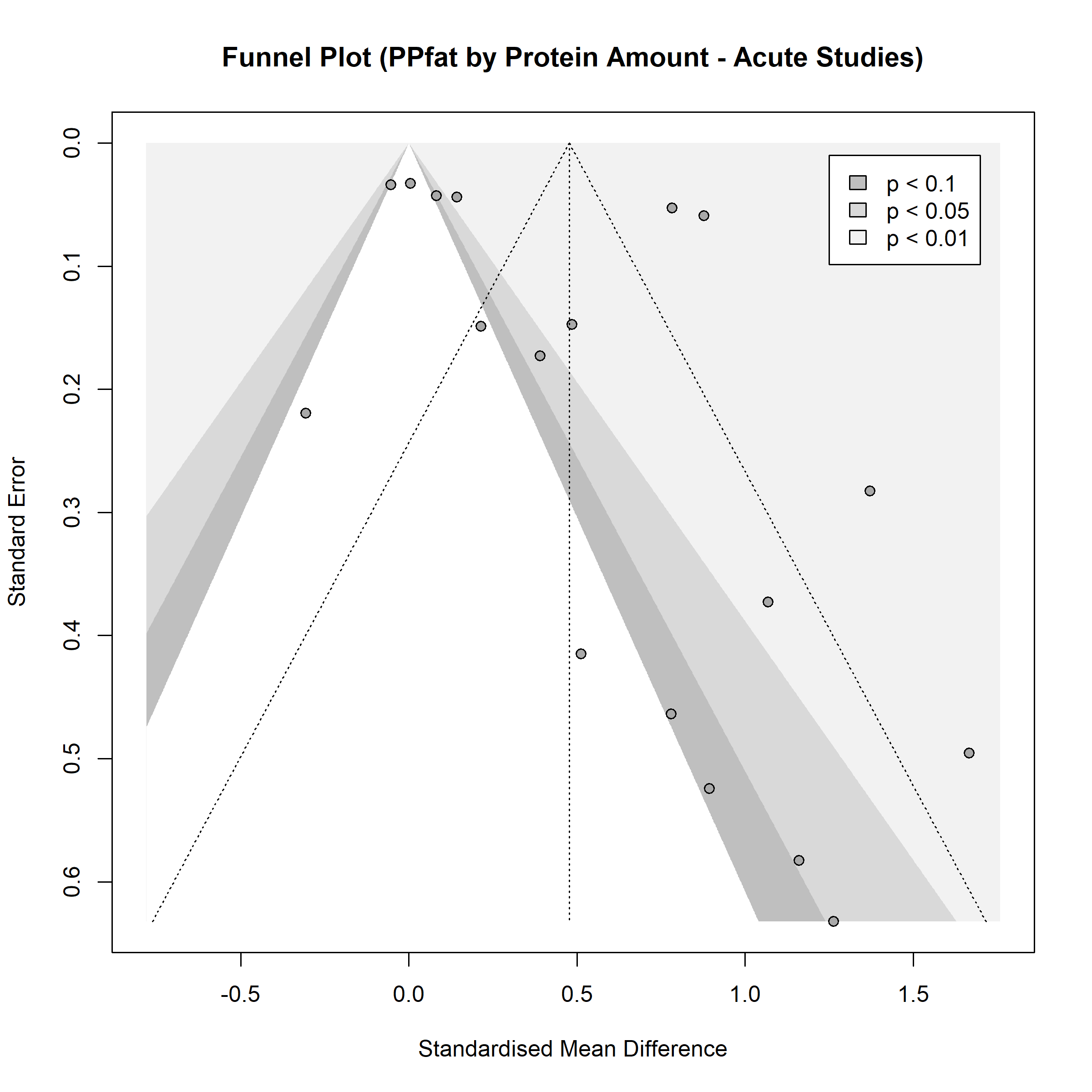

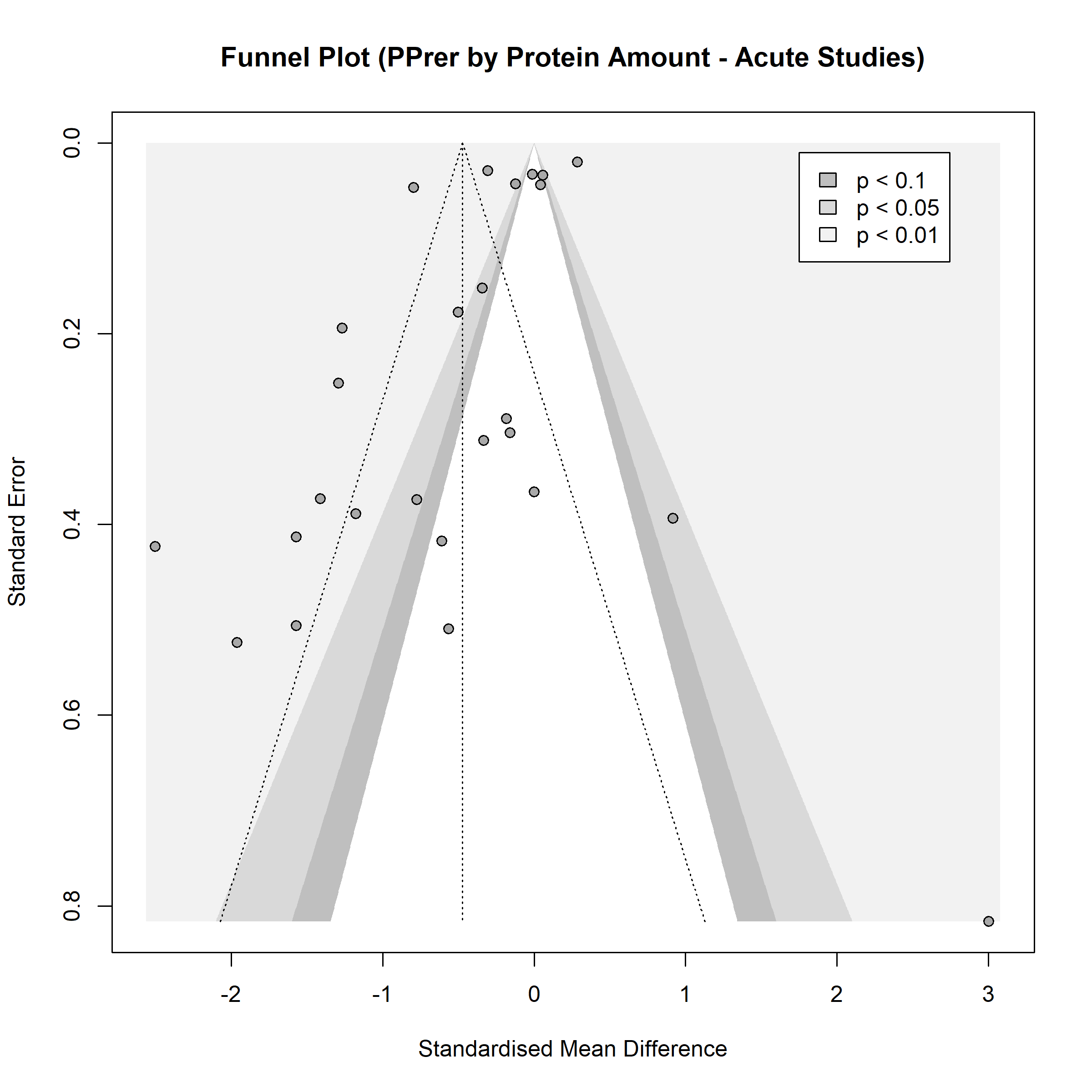


**Supplemental Figure 3.** Contour-enhanced funnel plot for analyses of the effect of chronic diets containing different amounts of protein on (A) diet induced thermogenesis (B) total daily energy expenditure, (C) resting energy expenditure, (D) postprandial carbohydrate oxidation, (E) postprandial fat oxidation, and (F) postprandial respiratory exchange ratio. Shaded regions represent varying levels of statistical significance.

A B C


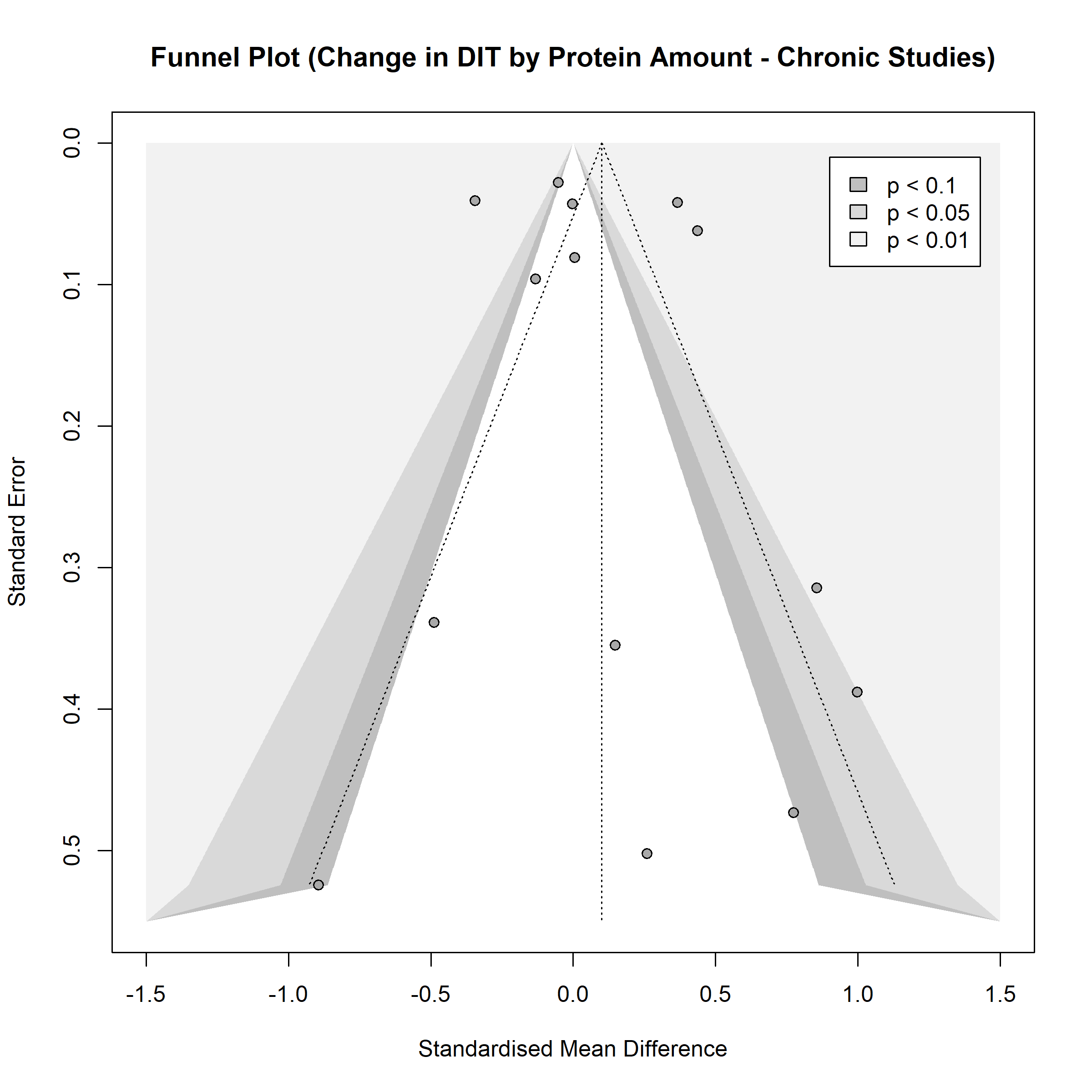

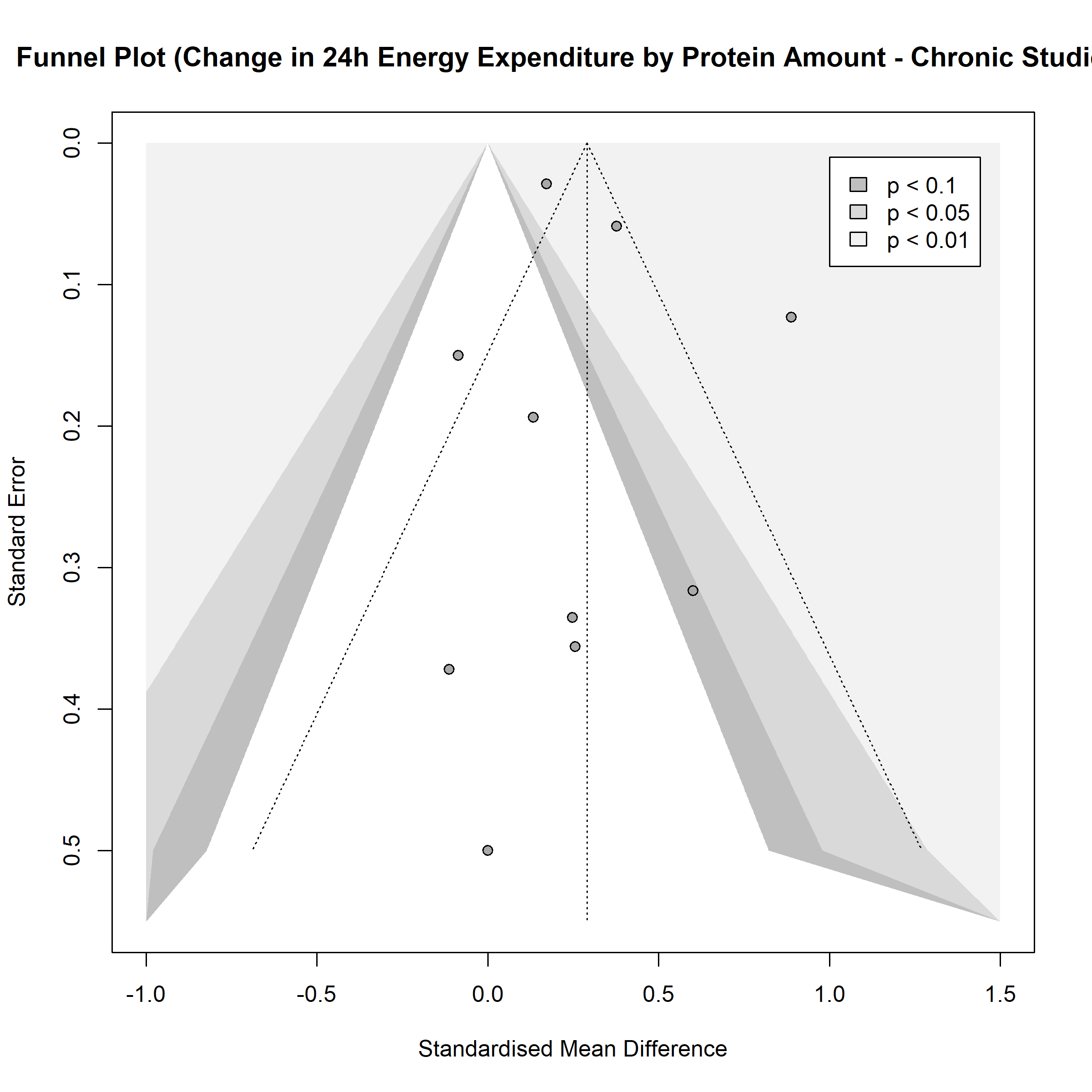

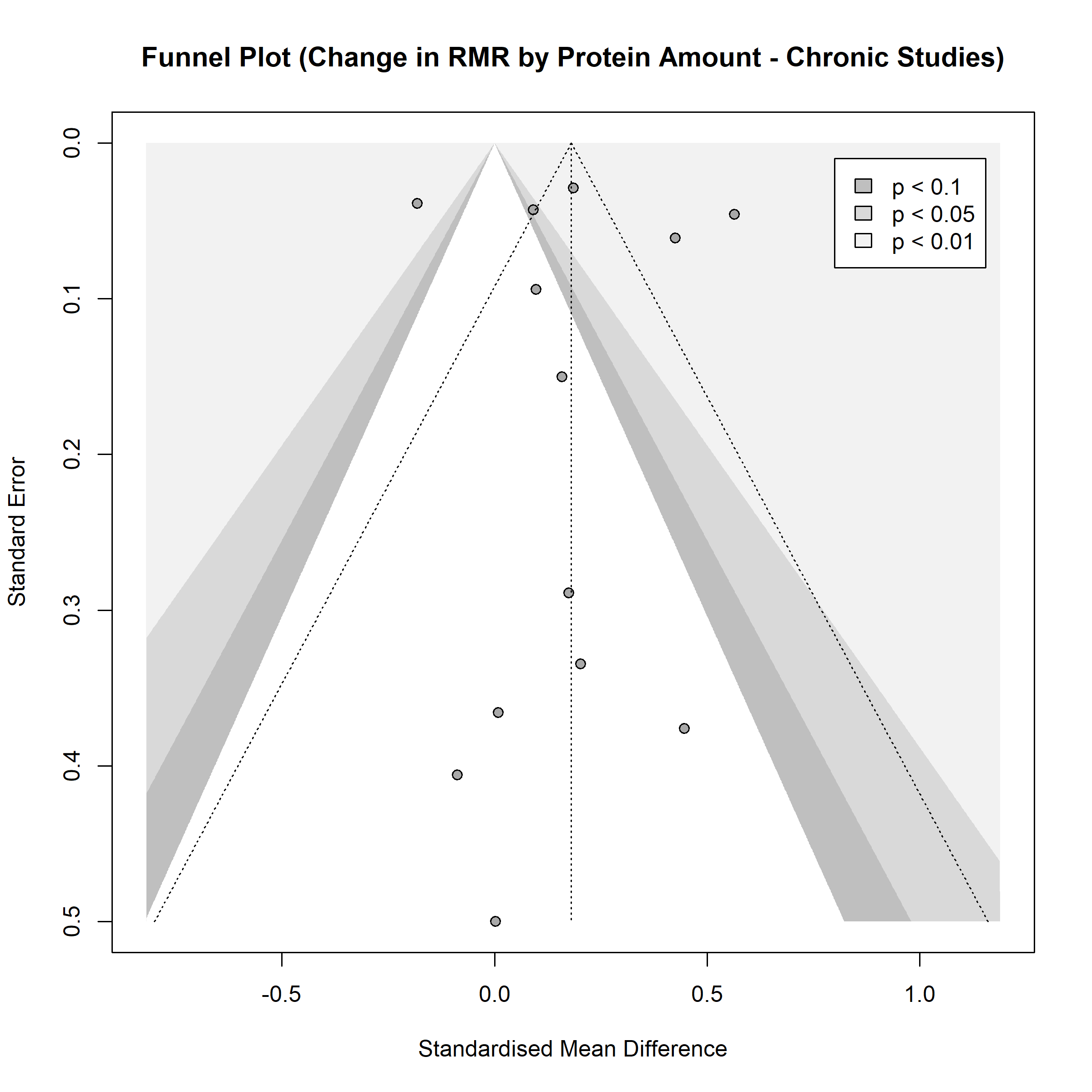


D E F


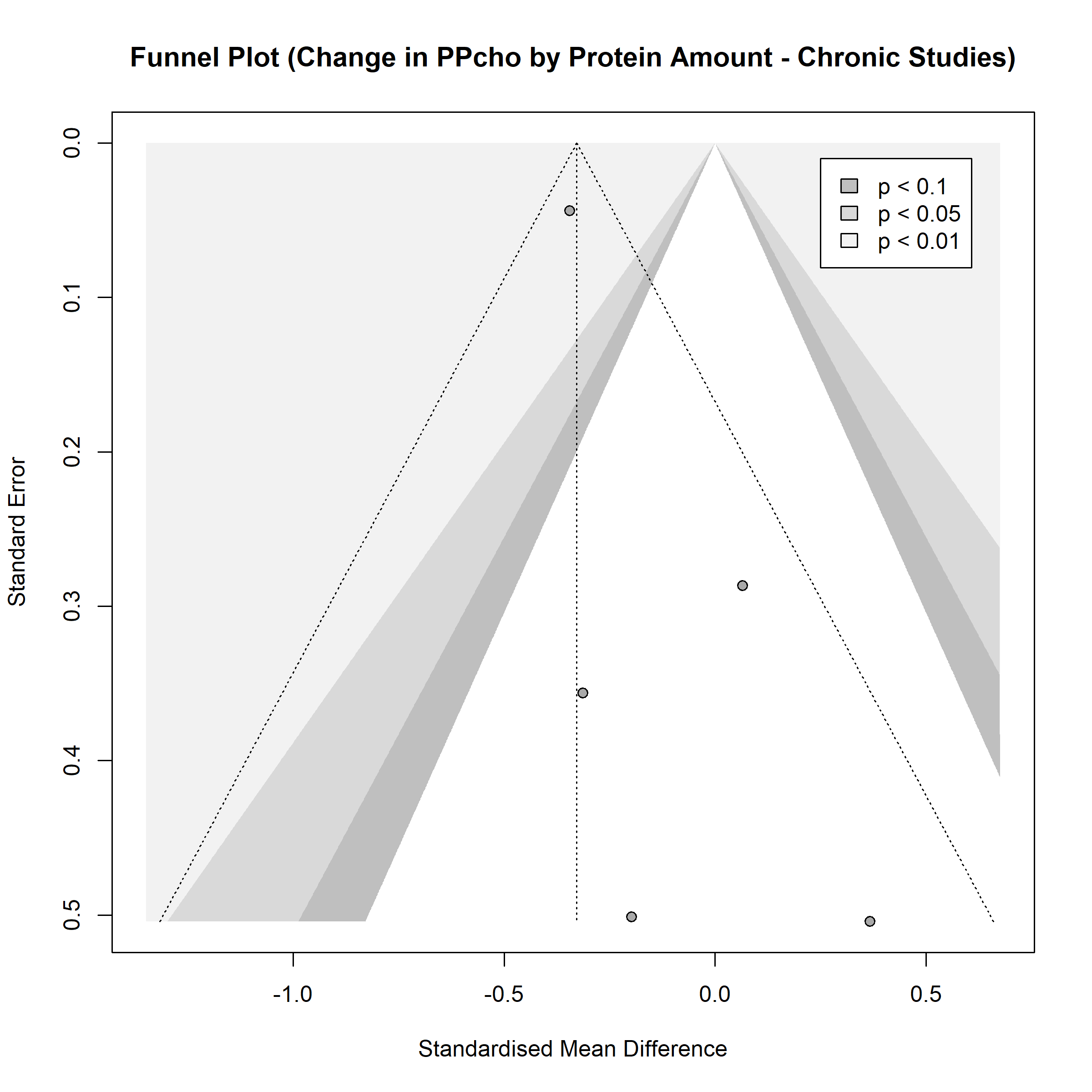

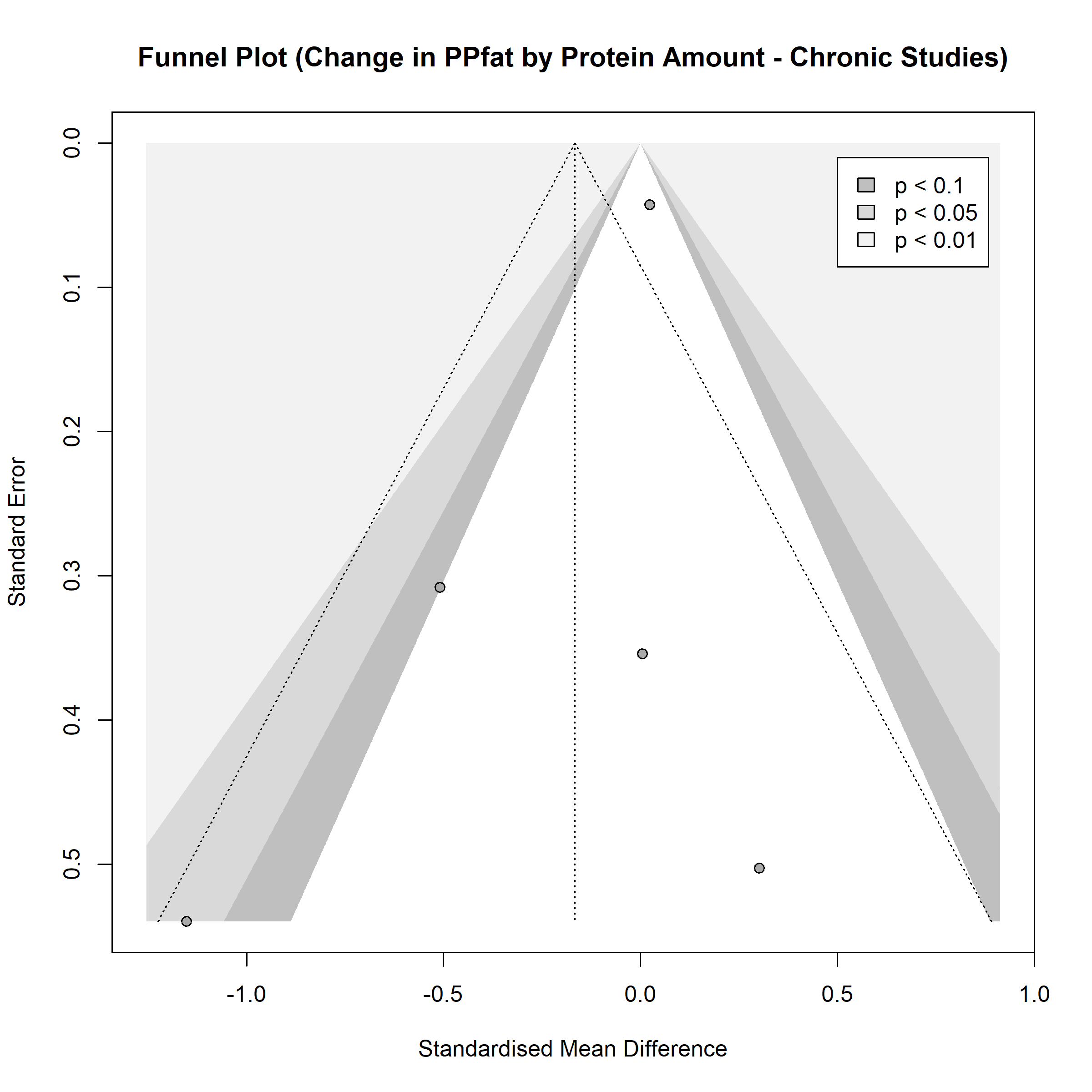

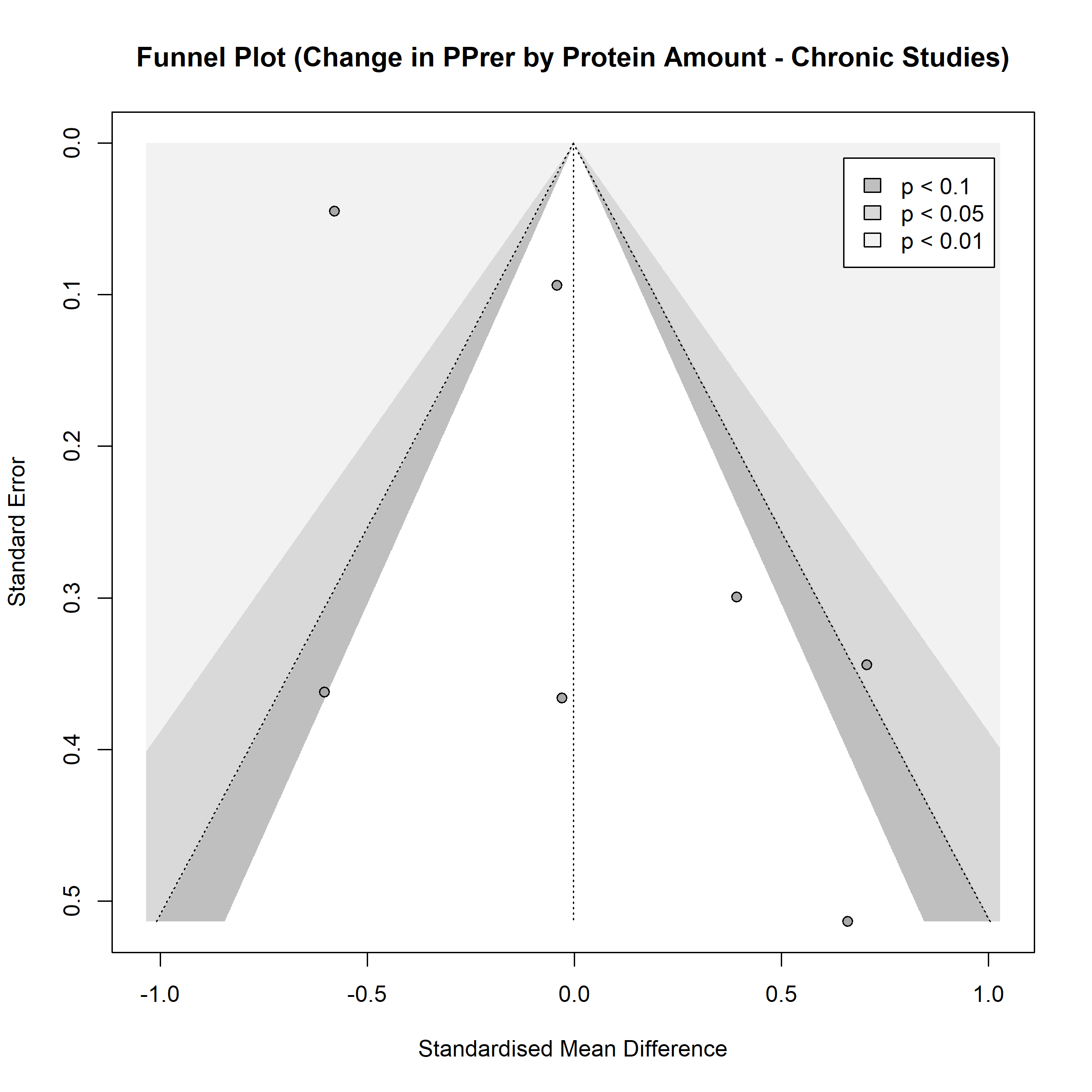


**Supplemental Figure 4.** Forest plots for the effect of chronic diets containing different amounts of protein on total daily energy expenditure in the subgroups for (A) parallel and (B) crossover study designs and resting energy expenditure in the subgroups for (C) parallel and (D) crossover study designs.

A B


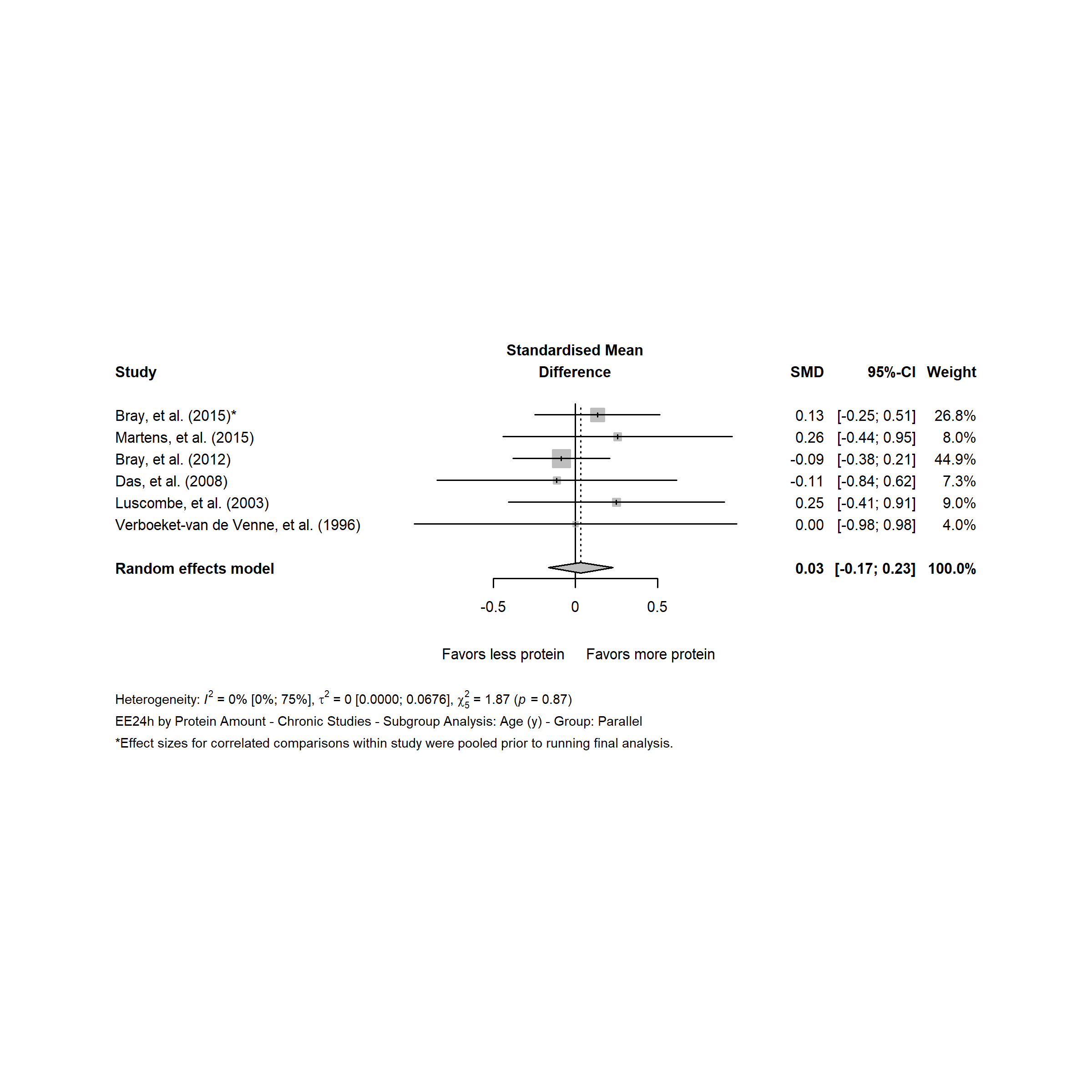

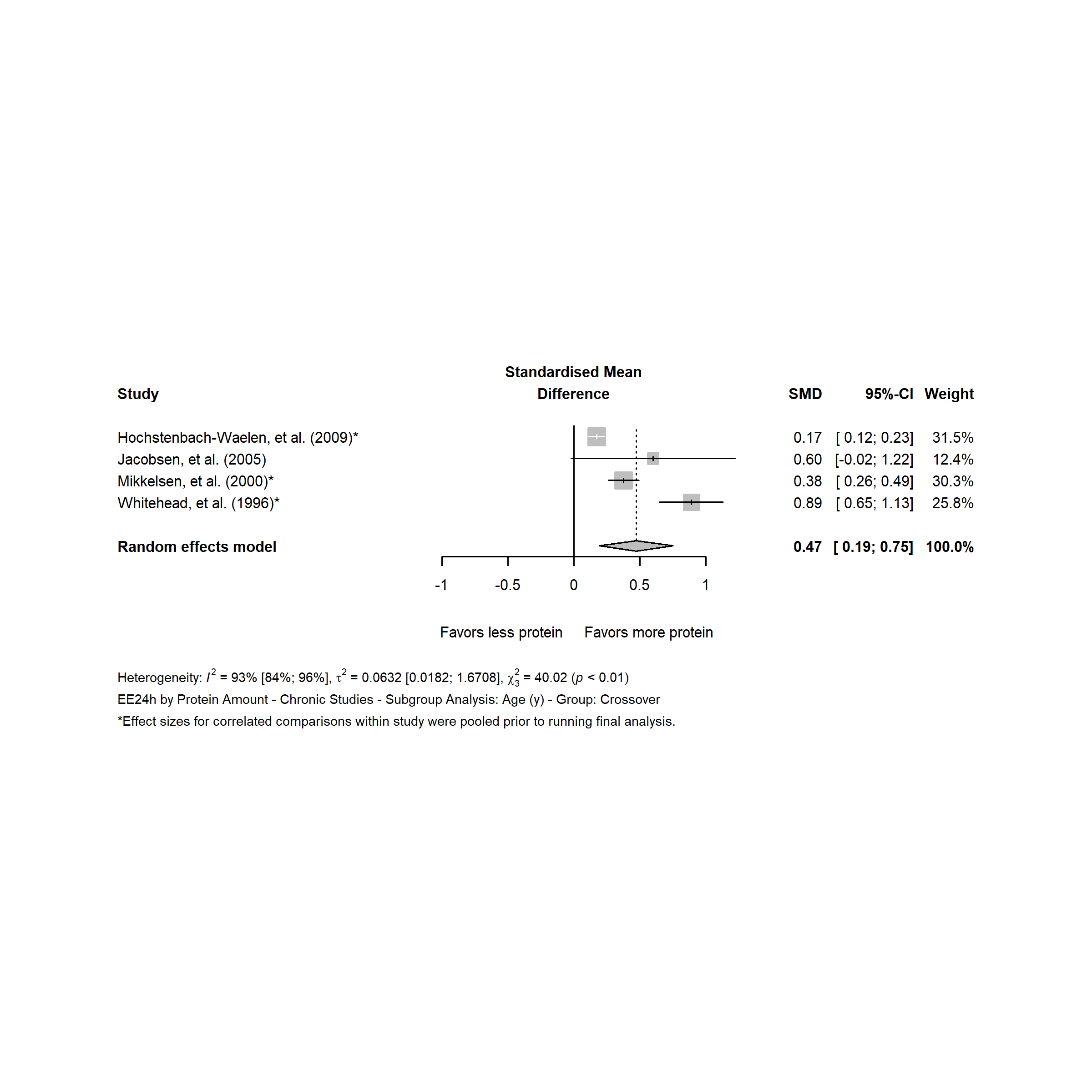


C D

**
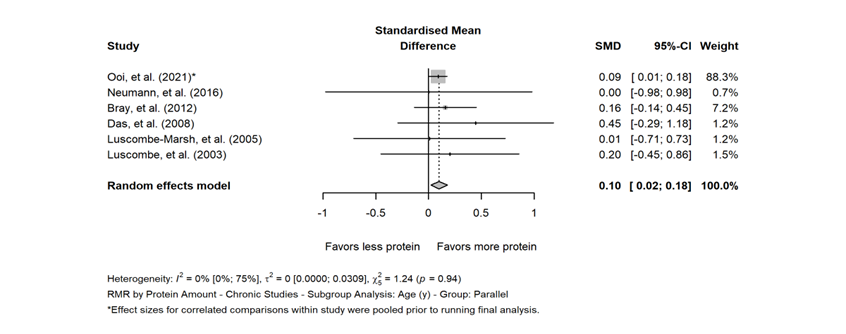
** **
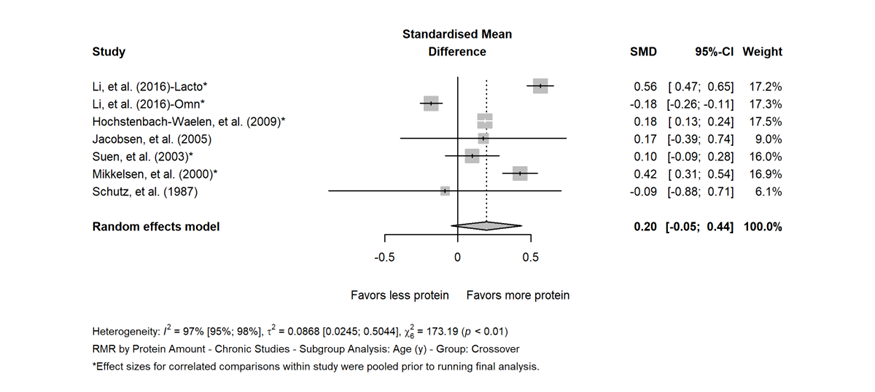
**
